# Diversity and putative interactions of parasitic alveolates belonging to Syndiniales at a coastal Pacific site

**DOI:** 10.1111/1758-2229.13138

**Published:** 2023-02-13

**Authors:** Maitreyi Nagarkar, Brian Palenik

**Affiliations:** ^1^ Scripps Institution of Oceanography University of California San Diego La Jolla California USA; ^2^ Present address: 26 Martin Luther King Dr West Cincinnati, OH USA

## Abstract

The dinoflagellate lineage Syndiniales currently consists entirely of parasitic species that fall into five well‐supported clades. Environmental sequencing studies worldwide have found an abundance of Syndiniales in a variety of marine ecosystems, but very little is known about the majority of Syndiniales species including two entire clades which have only been observed in sequence data. Syndiniales are known to have a wide range of hosts, but only a few dozen interactions have been confirmed through observation of actual infections. Here, we describe the diversity of Syndiniales found at the Scripps Institution of Oceanography pier over the course of a year based on 18S sequencing. We find Syndiniales to be the most species (amplicon sequence variant)‐rich taxonomic group and for its members to be present and abundant throughout the year. We used several analytical techniques to identify potential parasite–host interactions which we were then able to visualize over time. Using mock communities and size fractionation of seawater, we suggest that the majority of Syndiniales sequences that are found in environmental studies belong to the free‐living dinospore stage rather than representing active infections.

## INTRODUCTION

The parasitic group Syndiniales has been increasingly recognized due to its abundance and ubiquity in sequencing studies at numerous sites globally (Coats & Park, 2002; de Vargas et al., 2015; Guillou et al., 2008; Park et al., 2013; Skovgaard et al., 2005, 2008). This is believed to be a monophyletic and entirely parasitic lineage with several clades consisting entirely of uncultured species (Guillou et al., 2008; Moon‐van der Staay et al., 2001). These marine parasites have a wide variety of hosts, including other protist parasites, ciliates, radiolarians, tintinnids, dinoflagellates (including harmful bloom formers), fish eggs, copepods and crabs (Coats, 1999; Guillou et al., 2008; Jung et al., 2016; Shields, 1994; Skovgaard et al., 2005; Stentiford & Shields, 2005). The Syndiniales comprise at least five major marine alveolate (MALV) groups, referred to interchangeably as MALV I–V, Syndiniales Groups I–V, or, in the PR2 database Dino‐Group I–V (Chambouvet et al., 2011; Guillou et al., 2008; Guillou et al., 2013). Syndiniales Group II is currently recognized as the most diverse clade and contains some of the most studied species, *Amoebophrya* spp; most of these have been identified as parasites of various dinoflagellates. Group I includes *Ichthyodinium*, a parasite of fish eggs, and other species known to infect ciliates, Group IV contains members that infect other metazoans (such as crabs and copepods), including *Hematodinium*, and *Syndinium*, and Groups III and V currently consist entirely of environmental sequences, but form well‐supported clades (Guillou et al., 2008). A few Syndiniales species have been studied more closely in lab‐based experiments, demonstrating a broad range of hosts but a fairly similar life cycle. An example of an *Amoebophrya* life cycle in species that infect dinoflagellates shows three stages: dinospore, trophont, and vermiform (Coats & Park, 2002). The dinospore is an infective dispersal stage. (Siano et al., 2011); once it has infected the host cytoplasm or nucleus, it enters the trophont stage and replicates its own nucleus. Finally, it evacuates the host by rupturing its membrane and releases short‐lived vermiforms that complete cytokineseis to become biflagellated dinospores, which are much smaller than most known Syndiniales hosts (Coats & Bockstahler, 1994).

There is evidence that parasitism may play a role in the decline of dinoflagellate blooms. This has even been suggested as a means of controlling harmful algal blooms since many toxic dinoflagellates have been identified as Syndiniales hosts (Taylor, 1968). In the Penzé estuary in France, Chambouvet et al. (2008) found successive blooms and declines of four different dinoflagellate species, *Heterocapsa rotundata*, *Scrippsiella troichoidea*, *Alexandrium minutum* and *Heterocapsa triquetra* over the course of several weeks. They were able to visually track Syndiniales infections using fluorescent probes specific to Syndiniales Group II. Sequencing marker genes at different time points, revealed the prevalence of different Syndiniales species during or shortly lagging each of the four dinoflagellate blooms. Montagnes et al. (2008) found that the introduction of parasitism in models of plankton dynamics at this site allowed models to better reflect bloom dynamics. In the Californa Current Ecosystem, Mazzillo et al. (2011) found that large blooms of the dinoflagellate *Akashiwo sanguinea* were associated with low *Amoebophrya* prevalence, while non‐red tide periods contained a higher percentage of infected *Akashiwo* cells. One of the years with an especially long red tide, no *Amoebophrya* were detected, which suggests an important role for these parasites in maintaining *A. sanguinea* populations at a low level. It is possible a similar phenomenon occurs at the Scripps Institution of Oceanography (SIO) pier; in 2011, the date of the largest *A. sanguinea* pier bloom corresponded with the lowest relative abundance of *Amoebophrya* and Syndiniales sequences (Nagarkar et al., 2018). This bloom could have been a result of *A. sanguinea* growth becoming greater than its mortality rate due to some factor leading to *Amoebophrya* decline. There is evidence that ciliates graze on *Amoebophrya* dinospores, reducing their infection rates on dinoflagellates such as *A. sanguinea* (Johansson & Coats, 2002).

Known parasites comprised more than half of both the richness and abundance of sequences within the piconanoplankton collected in the global TARA expedition, which sampled at 68 stations across the global oceans (de Vargas et al., 2015). At our own site, the Scripps Institution of Oceanography Pier, Syndiniales sequences comprised as much as 11% of sequences at a given sampling point (Nagarkar et al., 2018). However, it is unclear whether their abundance in sequence data truly translates to environmental abundance. This is greatly complicated by the known variation in18S rRNA copy number in eukaryotes and particularly dinoflagellates (Gong & Marchetti, 2019), and the fact that their different life stages include numerous nuclei when inside a host, as well as dinospores which have been found in some species to survive for up to 2 weeks in their free‐living state, but may not ultimately infect a new host. Each of these life stages consists of different cell sizes and survives for a different amount of time. For one species of *Amoebophrya* that was observed to infect *Gymnodinium sanguineum*, dinospores were smaller than 10 μm in size and survived independently on the order of days; trophonts were multinuclear and reached up to 20 μm, with infections that also lasted on the order of days; vermiforms appear as long connected cell chains, with a length possibly in the hundreds of microns, but are released as dinospores within minutes (Coats & Bockstahler, 1994; Coats & Park, 2002).

Recent studies have used co‐occurrence analyses on a large geographical scale to infer parasite–host pairs. For example, the TARA Oceans study, using co‐occurrence matrices made from OTU abundance data at numerous sites, suggested that parasitism was the most abundant type of taxon–taxon interaction (Lima‐mendez et al., 2015). A majority of these interactions involved the Syndiniales, especially clades I and II. Other studies have encountered unexpected abundances and diversity of Syndiniales in a variety of environments, including the Antarctic (Cleary & Durbin, 2016), the Baltic Sea (Majaneva et al., 2012) and Korean coastal waters (Kim et al., 2008). However, we know marine communities to be dynamic over time, especially at coastal sites, and geographical co‐occurrence should be supplemented with time‐series studies at a given site which may be able to validate or reveal new interactions. In this study, we use high‐frequency environmental sequencing at this single site to describe the dynamics of the eukaryotic community as a whole and attempt to identify individual parasite–host interactions that might be ecologically relevant.

## EXPERIMENTAL PROCEDURES

### Sample collection and DNA extraction

We sequenced 87 seawater samples collected from the Scripps pier along with several additional replicates and mock communities. For sampling, surface seawater was collected by bucket at the end of the Scripps pier (32^o^87′N, 117^o^26′W) at approximately 9:00 AM PST on every Monday and Thursday (with one exception, 4 July) in 2016. About 500 ml was filtered in triplicate onto a 47 mm 0.2 μm Supor filter (Pall) unless otherwise indicated. Filters were wrapped in aluminium foil and stored at −80°C until the time of DNA extraction. DNA extractions were conducted in sets of two to four filters. Filters were cut into small pieces on a clean surface and placed in 2 ml tubes with 560 μl TE (50 mM Tris, 20 mM EDTA) and 80 μl of 100 mg/ml lysozyme. *Schizosaccharomyces pombe* DNA (ATCC culture 24843) was spiked in at this point for some of the samples as a potential means of calculating recovery, but its sequences were removed from further analysis due to inconsistent recovery. Samples were incubated at 37°C for half an hour. We then added 80 μl 10 mg/ml Proteinase K and 80 μl 10% SDS and incubated at 55°C for 2.5 h. Finally, we added 10 μl 10 mg/ml RNAse A and incubated at 37°C for another half hour. DNA was extracted in an equal volume of phenol:chloroform:isoamyl alcohol twice and then once more with chloroform:isoamyl alcohol. Finally, we eluted DNA from the aqueous phase using the Qiagen DNEasy Blood and Tissue kit (Qiagen) according to the manufacturer's instructions. DNA was stored at −20°C until further use.

### Library preparation

Due to cost constraints, DNA libraries were prepared using two different methods, but both used DNA extracts obtained using the method described above. For approximately half the samples, triplicate 25 μl PCRs were performed on each sample using the Euk_1391F (5′‐GTACACACCGCCCGTC‐3′) and EukBr (5′‐TGATCCTTCTGCAGGTTCACCTAC‐3′) primers with single‐index barcodes on the forward primers. Reactions used 1 μl of each primer and 10 μl of GoTaq HotStart Master Mix (Promega). The triplicate reactions were combined and DNA concentration was measured using a Qubit fluorometer. Unincorporated nucleotides were removed using the ExoSAP‐IT cleanup kit (Thermo Fisher Scientific) according to the manufacturer's instructions. All sample DNA was pooled into a single tube with approximately 220 ng per sample, and the pooled sample was cleaned with Agencort AMPure beads (Beckman Coulter) according to the manufacturer's protocol. This sample was sent to the IGM for paired‐end 150 sequencing on the Illumina MiSeq. The remaining samples were sent to RTL Genomics for sequencing and processed according to their protocols (Supporting Information). A smaller subset of samples was sent to both facilities to evaluate whether results sequenced with slightly different methods were comparable.

### Data analysis

Sequences were assigned to ASVs (amplicon sequence variants) using deblur (Amir et al., 2017), with default parameters and a trim size of 155. Resulting ASVs were classified using BLCA (Gao et al., 2017) against the PR2 database (Guillou et al., 2013). ‘Unclassified’ sequences were examined with BLAST Subsequent analyses were conducted in R using the phyloseq (Mcmurdie & Holmes, 2013) package. Hierarchical clustering was used to ensure that replicate samples (sequenced in the two different facilities) clustered together relative to all samples. All sequencing data were reported and analysed together.

Correlation analyses were conducted using SparCC (Friedman & Alm, 2012). *S. pombe* sequences were removed before all analyses. Before running SparCC, metazoan sequences, unclassified sequences, and ASVs with fewer than 100 sequences were eliminated, along with ASVs with greater than 60 absences (zeros) in the data set (of 87 samples). After these cutoffs, a total of 1043 ASVs were run against one another using SparCC to find significant correlations. Interacting pairs with *r* = 0.6 or greater were reported unless otherwise specified.

## RESULTS

### Coupling of microscopic observations and amplicon data can reveal putative parasite–host pairs

**FIGURE 1:**
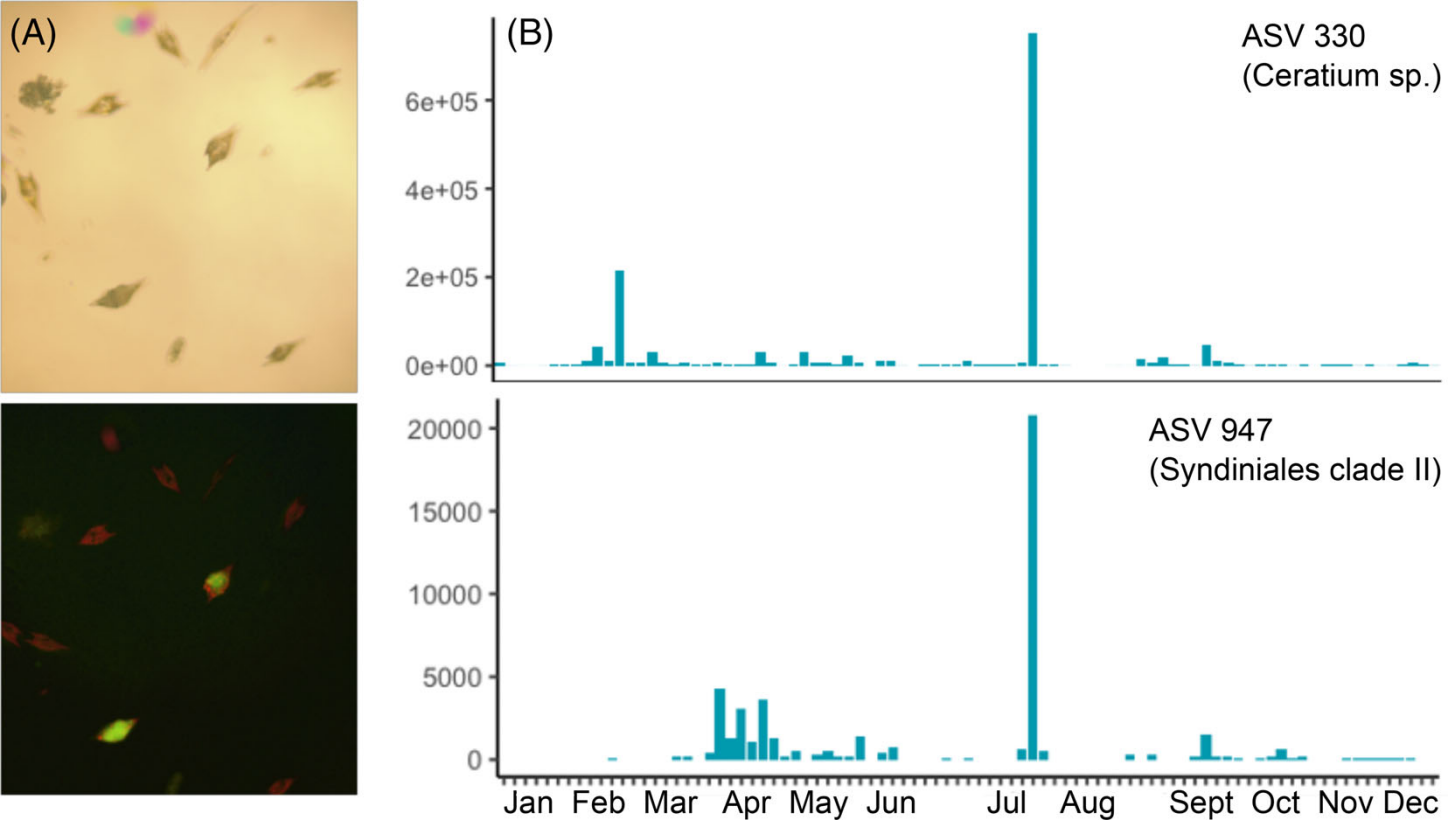
(A) Microscopic observation of seawater from March 2018 bloom with many *Ceratium* sp. present in bright field (top) and under blue excitation light (bottom). Red fluorescence is the chloroplasts and green fluorescence indicates *Amoebophrya* infection. These *Ceratium* are typically 50 μm in cell width. (B) Read abundance of the putative ASVs in the 2016 time series representing the dinoflagellate host (*Ceratium* ASV 330) and parasite *Amoebophrya* (ASV 947) (based on sequence analysis of the 2018 seawater sample). ASV, amplicon sequence variant.

In March 2018, a large diatom and *Ceratium* dinoflagellate bloom occurred off the SIO pier. Ceratium were visibly infected with parasites based on *Amoebophrya's* characteristic green autofluorescence (Figure 1). We isolated DNA from a water sample and using amplicon sequencing found a *Ceratium* ASV (later annotated as ASV 330) and Syndiniales clade II ASV (later ASV 947) that were highly abundant. The Syndiniales clade II ASV had a 100% match on BLAST with *Amoebophrya ex. Ceratium tripos* (Accession no. AY208892). Thus microscope analyses can be used to monitor for events where amplicon analyses could be conducted to identify likely ‘bloom’‐level parasite interactions. To monitor lower‐level interactions of common pier community members, we chose to apply amplicon sequencing to a year (2016) of available samples.

### The eukaryotic community at the SIO pier over time

**FIGURE 2:**
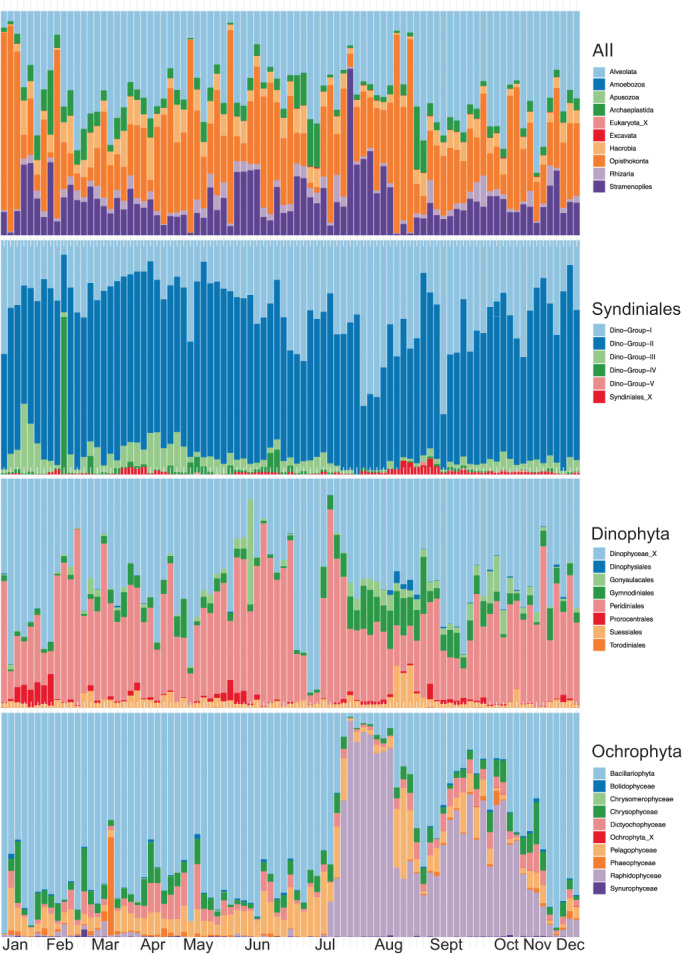
Eukaryotic community at the SIO pier over the course of 2016. Metazoan sequences, unclassified sequences, and *Schizosaccharomyces pombe* sequences were removed, and replicate samples were pooled.

From 87 samples containing 5,181,108 sequences, we found there were 5632 ASVs at the pier in 2016 (Table S1). As expected, there were fluctuations in the community composition at both broad and fine taxonomic levels (Figure 2). Several phenomena were immediately observable in these sequence data including a dinoflagellate bloom in early July followed by a raphidophyte bloom starting in July that was corroborated in observational data (Figure 2).

**FIGURE 3:**
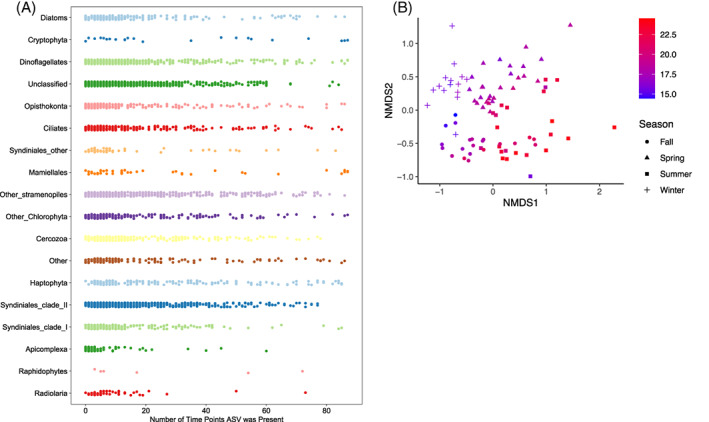
(A) Number of time points ASVs were detected at, grouped by taxonomic level. Each point represents one ASV. Unclassified sequences were not removed and may include bacterial and archaeal sequences. ‘Other’ refers to all other taxa not included in the other groups. (B) non‐metric multidimensional scaling plot of all SIO pier samples, coloured by temperature. Shapes represent the calendar season of the sample. ASV, amplicon sequence variant.

Only six ASVs were present in every single sample: one cryptophyte (ASV 4, *Teleaulax* sp.), two dinoflagellates (ASV 9, *Heterocapsa* sp., and ASV 145, *Biecheleria* sp.), and three chlorophytes, (ASV 25, *Bathycoccus* sp., ASV 44, *Micromonas pusilla*, and ASV 34, *Pyramimonas* sp.). Classifications provided here were the top BLAST hit for each ASV sequence. The vast majority of ASVs were present at fewer than half the time points, with certain groups like apicomplexans and radiolarians comprised mostly of ASVs that were detected on fewer than 10 time points (Figure 3A). Among the 50 ASVs that were present for greater than 90% of time points, four belonged to the Syndiniales—ASVs 14, 76, 233, and 707. ASV 14 was classified in Syndiniales Group III and the others were in Group I. An ordination analysis of all time points reveals there to be a seasonal structuring of the community (Figure 3B).

### Syndiniales sequences are highly diverse and abundant at the pier

There were 997 total Syndiniales ASVs, making it the most diverse taxonomic group that was classified in our pipeline. There were hundreds more Syndiniales ASVs than any other ubiquitous taxa, including the stramenopiles, dinoflagellates, ochrophytes, metazoans, chlorophytes, cercozoans and haptophytes (Figure 2A). Syndiniales sequences were not typically the most abundant in samples, ranging from 2% to 20% of total sequences during a given time point; the greatest proportion of total sequences was usually from other dinoflagellate taxa (Figure 2). Syndiniales clades I and II were the most abundant, with clades III, IV and V present at lower levels—though ASV 14, of clade III, was present nearly all the time. The Syndiniales proportion of reads was greatest in the summer dates, but individual ASVs had distinct times of year when they were present, with very few present all throughout the year (Figure 2A).

### Identification of putative Syndiniales–host interactions

Given that currently characterized members of Syndiniales spend a portion of their life cycle within their host, it is likely that host–parasite pairs would co‐occur. As a proof‐of‐concept, we first explored whether sequences corresponding to the Syndiniales–*Karlodinium* pair that we maintained in culture (Bai et al., 2007, obtained from *T. Bachvaroff*) appeared, and co‐occurred, in our pier samples. Both sequences did indeed co‐occur, but were only present abundantly on a single date (9 June 2016), so their relationship could not be tracked temporally in this dataset.

**FIGURE 4:**
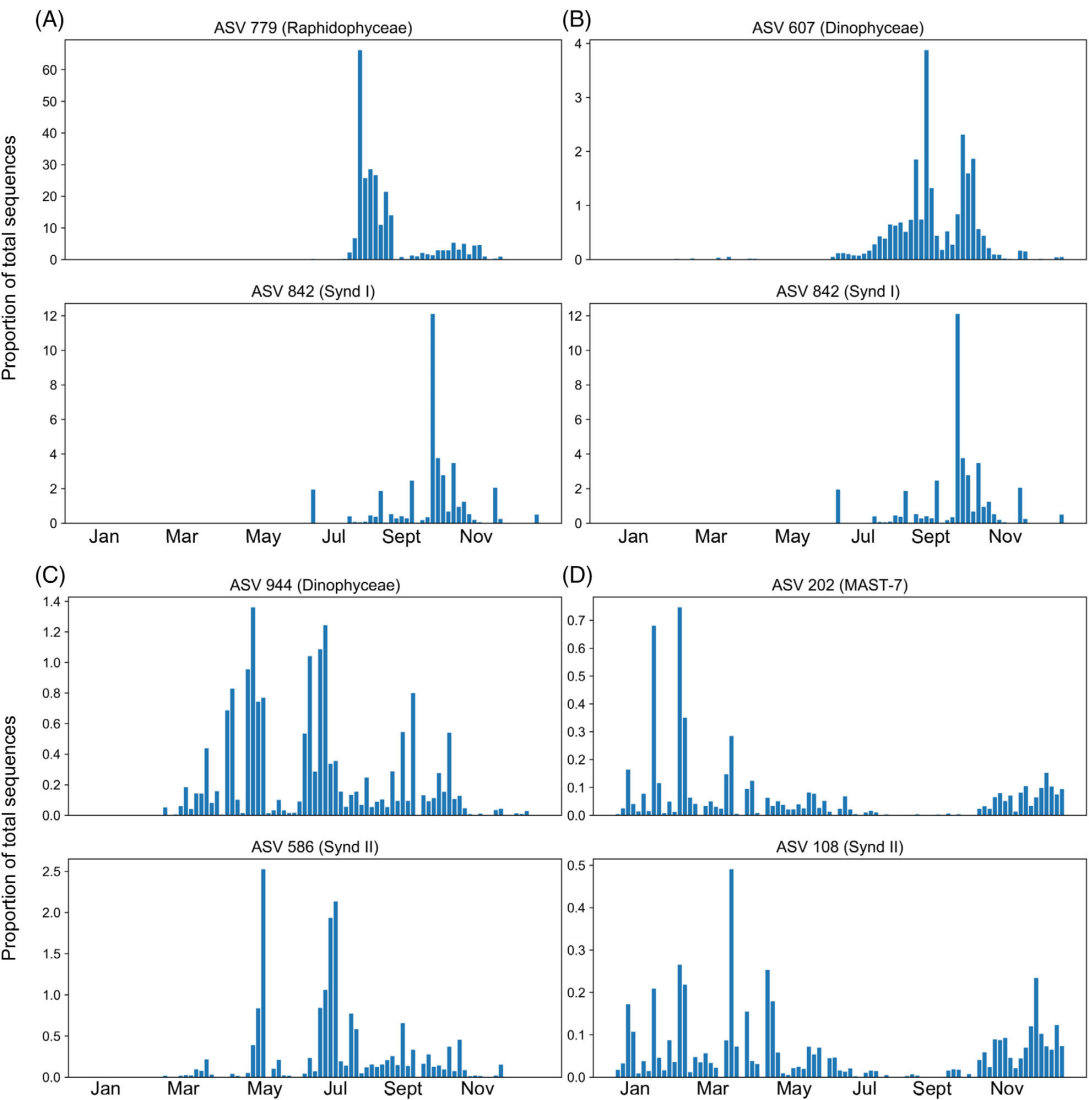
Proportion of sequence reads over time represented by the four most highly correlated pairs of ASVs (using SparCC) that included a member of Syndiniales. (A) *r* = 0.78, (B) *r* = 0.75, (C) *r* = 0.74 and (D) *r* = 0.74. ASV, amplicon sequence variant.

We conducted a co‐occurrence analysis using SparCC (Friedman & Alm, 2012) and found 664 significant (*p* < 0.01) correlations with *r* > 0.6, of which 135 involved a Syndiniales ASV (Table 1). There were significant correlations among Syndiniales ASVs and a wide variety of other ASVs, including ciliates (10), raphidophytes (8), diatoms (20), dinoflagellates (21), chlorophytes (21), cercozoans (10) and other members of the Syndiniales (11). Four examples of highly correlated Syndiniales–host pairs are provided in Figure 4A–D. Note that SparCC correlations were obtained based on proportions. A full list of highly correlated pairs is provided in Table S2.

**TABLE 1:** Significant (*p* < 0.005) correlations identified by SparCC between Syndiniales ASVs and other ASVs

Syndiniales	ASV	Tax	ASV 2	r
Dino‐Group‐I	ASV_1035	Chlorellales	ASV_983	0.63
		Dino‐Group‐II	ASV_1093	0.64
		Dino‐Group‐II	ASV_929	0.62
Dino‐Group‐I	ASV_1317	Dinophyceae_X	ASV_607	0.62
		Telonemia_XX	ASV_951	0.61
		Raphidophyceae_X	ASV_779	0.6
		Syndiniales_X	ASV_950	0.62
Dino‐Group‐I	ASV_163	Bacillariophyta_X	ASV_36	0.64
		Bacillariophyta_X	ASV_2	0.62
Dino‐Group‐I	ASV_195	Prorocentrales	ASV_32	0.65
		Cryomonadida	ASV_150	0.62
Dino‐Group‐I	ASV_2160	Bacillariophyta_X	ASV_28	0.68
		Dinophyceae_X	ASV_1570	0.66
		Cryomonadida	ASV_1711	0.65
		Bacillariophyta_X	ASV_3	0.61
		Chlorellales	ASV_35	0.61
Dino‐Group‐I	ASV_2204	Cryomonadida	ASV_1711	0.71
		Filosa‐Thecofilosea_X	ASV_708	0.64
		Bacillariophyta_X	ASV_3	0.64
		Chlorellales	ASV_35	0.63
		Dino‐Group‐II	ASV_237	0.67
Dino‐Group‐I	ASV_707	Prymnesiales	ASV_95	0.61
Dino‐Group‐I	ASV_842	Telonemia_XX	ASV_951	0.73
		Dinophyceae_X	ASV_961	0.72
		Dinophyceae_X	ASV_1037	0.71
		Pyramimonadales_X	ASV_900	0.71
		Tintinnida	ASV_933	0.7
		Gymnodiniales	ASV_1014	0.69
		Oomycota_X	ASV_1077	0.67
		Strombidiida	ASV_2086	0.66
		Choreotrichida	ASV_967	0.65
		Telonemia_XX	ASV_1054	0.65
		Strombidiida	ASV_848	0.63
		MAST‐3	ASV_1041	0.63
		Raphidophyceae_X	ASV_779	0.78
		Dinophyceae_X	ASV_607	0.75
		Bacillariophyta_X	ASV_67	0.61
		Syndiniales_X	ASV_950	0.72
		Dino‐Group‐II	ASV_898	0.65
		Dino‐Group‐II	ASV_996	0.62
Dino‐Group‐I	ASV_880	Raphidophyceae_X	ASV_779	0.64
		Chlorarachnida	ASV_837	0.61
Dino‐Group‐I	ASV_964	Dinophyceae_X	ASV_1037	0.65
		MAST‐3	ASV_1041	0.62
		Raphidophyceae_X	ASV_779	0.68
		Telonemia_XX	ASV_951	0.65
Dino‐Group‐II	ASV_1013	Gymnodiniales	ASV_1014	0.65
		Raphidophyceae_X	ASV_779	0.64
		Crustacea	ASV_877	0.62
		Dinophyceae_X	ASV_961	0.6
Dino‐Group‐II	ASV_1057	Gymnodiniales	ASV_908	0.71
		Raphidophyceae_X	ASV_779	0.69
		Tintinnida	ASV_933	0.67
		Phaeocystales	ASV_1020	0.61
		Dinophyceae_X	ASV_607	0.61
Dino‐Group‐II	ASV_108	MAST‐7	ASV_202	0.74
		MAST‐4	ASV_218	0.61
		Mamiellales	ASV_25	0.71
		Mamiellales	ASV_40	0.64
		Pelagomonadales	ASV_22	0.64
		Mamiellales	ASV‐15	0.61
Dino‐Group‐II	ASV_109	Dinophyceae_X	ASV_11	0.61
		Mamiellales	ASV_25	0.61
Dino‐Group‐II	ASV_1271	Raphidophyceae_X	ASV_779	0.62
		Tintinnida	ASV_933	0.61
Dino‐Group‐II	ASV_182	Cryomonadida	ASV_1711	0.63
		Chlorellales	ASV_35	0.67
		Bacillariophyta_X	ASV_28	0.65
		Bacillariophyta_X	ASV_3	0.64
Dino‐Group‐II	ASV_237	Cryomonadida	ASV_1711	0.69
		Bacillariophyta_X	ASV_28	0.73
		Bacillariophyta_X	ASV_3	0.7
		Chlorellales	ASV_35	0.65
		Bacillariophyta_X	ASV_26	0.64
		Bacillariophyta_X	ASV_2	0.64
		Pelagomonadales	ASV_58	0.61
		Cryomonadida	ASV_150	0.61
Dino‐Group‐II	ASV_249	Pezizomycotina	ASV_204	0.61
		Bacillariophyta_X	ASV_137	0.61
Dino‐Group‐II	ASV_291	Chlorellales	ASV_482	0.66
		Dino‐Group‐II	ASV_103	0.61
Dino‐Group‐II	ASV_328	Cryomonadida	ASV_1711	0.69
		Chlorellales	ASV_482	0.61
		Bacillariophyta_X	ASV_28	0.71
		Chlorellales	ASV_35	0.67
		Bacillariophyta_X	ASV_3	0.61
		Bacillariophyta_X	ASV_281	0.61
Dino‐Group‐II	ASV_57	MAST‐4	ASV_91	0.61
		Choreotrichida	ASV_31	0.61
		Bacillariophyta_X	ASV_2	0.61
		Dino‐Group‐II	ASV_103	0.62
		Dino‐Group‐II	ASV_195	0.61
Dino‐Group‐II	ASV_586	Dinophyceae_X	ASV_944	0.74
		Prymnesiales	ASV_629	0.67
		Chlamydomonadales	ASV_866	0.65
		Pelagophyceae	ASV_232	0.68
		Katablepharidales	ASV_560	0.62
		Dinophyceae_X	ASV_373	0.61
Dino‐Group‐II	ASV_592	Bacillariophyta_X	ASV_28	0.63
Dino‐Group‐II	ASV_625	Bacillariophyta_X	ASV_36	0.69
		Pelagomonadales	ASV_58	0.65
		Peridiniales	ASV_294	0.65
Dino‐Group‐II	ASV_929	Chrysophyceae_X	ASV_945	0.64
		Chlorellales	ASV_983	0.61
		Pyramimonadales	ASV_924	0.66
		Katablepharidales	ASV_560	0.65
		Chlorarachnida	ASV_772	0.64
		Dino‐Group‐II	ASV_942	0.67
Dino‐Group‐II	ASV_942	MAST‐1	ASV_993	0.61
		Pyramimonadales_X	ASV_924	0.63
Dino‐Group‐II	ASV_958	MOCH‐2	ASV_912	0.71
Dino‐Group‐II	ASV_973	Collodaria	ASV_2044	0.66
		Chrysophyceae_X	ASV_1443	0.6
Dino‐Group‐III	ASV_14	Mamiellales	ASV_25	0.69
		Katablepharidales	ASV_171	0.69
		Mamiellales	ASV_40	0.68
		Dictyochophyceae_X	ASV_133	0.62
Dino‐Group‐III	ASV_49	Dinophyceae_X	ASV_84	0.63
Syndiniales_X	ASV_950	Dinophyceae_X	ASV_1037	0.7
		Telonemia_XX	ASV_951	0.69
		Gymnodiniales	ASV_1014	0.69
		Strombidiida	ASV_2086	0.69
		Choreotrichida	ASV_967	0.68
		Dinophyceae_X	ASV_961	0.68
		Telonemia_XX	ASV_1054	0.66
		MAST‐3	ASV_1041	0.62
		Oomycota_X	ASV_1077	0.6
		Pyramimonadales_X	ASV_900	0.74
		Raphidophyceae_X	ASV_779	0.71
		Tintinnida	ASV_933	0.68
		Dinophyceae_X	ASV_607	0.67

We also analysed our data for the *Ceratium* ASV (330) and Syndiniales clade II ASV (947) discussed above. When tracked in the 2016 time series (Figure 1), these ASVs did co‐occur and indeed were both particularly abundant on the same date, but were not identified as an interacting pair given the SparCC correlation threshold of *r* = 0.6 (they had a significant but low SparCC correlation of *r* = 0.26). This illustrates the complementary strengths of microscopic and amplicon sequence data to find interacting pairs.

### Assessing the ability of sequence data to represent a protist community

We created several mock communities to assess whether a sample of known composition results in sequence data reflecting the known proportions. In the case of a mock community containing eight individual cultures, the dinoflagellates *Lingulodinium* and *Heterocapsa* were greatly overrepresented in the 18 S sequence data relative to all other members of the community (Figure S1A).

We also created mock communities containing only *Karlodinium* and *Amoebophrya*. We found that host sequences are over‐represented (over 75% of total sequences) even when the sample contains an equal number of *Karlodinium* cells and dinospore cells (Figure S1B). This is presumably due to a lower 18 S rRNA copy number in *Amoebophrya*.

### Separation of host cells and dinospores using size‐specific filtration

To examine whether the size fractionation of samples would provide information about the distribution of Syndiniales life stages (given the differential size of a trophont located inside a host cell and a free‐swimming dinospore), we compared a sample filtered directly on a 0.2 μm filter to a sample filtered on a 10 μm filter. We found that 10 μm filters were depleted in Syndiniales sequences as a proportion of total dinoflagellate sequences (Figure S2) suggesting that most Syndinales could be in the dinospore stage. This was also consistent with other filtration experiments in Figure S2.

### Other parasites at the SIO pier

In addition to the Syndiniales, our data showed the presence of other parasites. For example, ASV 253, ASV 552, ASV 292, ASV 134, ASV 2398, ASV 3364, ASV 2065, ASV 5459, ASV 1504 and ASV 800 are identified as related to *Paradinium*, a known parasite of copepods (Skovgaard and Daugbjerg, 2008). We also found 148 ASVs identified as Gregarines, also a diverse group of parasites (Leander et al., 2003). While we did not characterize these further, it is clear that parasites other than the Syndiniales are an active component of the marine microbial community at the SIO pier.

## DISCUSSION

We chose to explore the dynamics of Syndiniales populations at our site because of their previously suggested role in driving the population dynamics of their hosts (Chambouvet et al., 2008; Coats et al., 1996; Coats & Bockstahler, 1994), particularly dinoflagellates, which are known to be abundant and have large blooms at our site (Nagarkar et al., 2018). If abundance of rDNA sequences is any indication (as our mock communities containing *Amoebophrya* suggest), then it is clear that members of Syndiniales are a large component of marine microbial communities including at the SIO pier. Yet, relatively little is known about the ecological strategies or even simply the hosts of most Syndiniales species. Members of Syndiniales were found to have the greatest number of species interactions within numerous ecosystems in a large‐scale co‐occurrence‐based study (Lima‐mendez et al., 2015). By characterizing Syndiniales populations with time at a single site, we provide further insights into species–species interactions.

We obtained a large number and diversity of Syndiniales ASVs from SIO Pier samples. Many sequences appeared transiently for a few weeks or months but were absent the rest of the year. Members of Syndiniales did not ever constitute the largest proportion of ASVs in a given sample, but were consistently abundant (2%–20% of ASVs). Syndiniales were, however, the taxonomic group with greatest species (ASV) richness, with the majority of ASVs falling within clade II. While this remarkable diversity is unsurprising given the worldwide ubiquitous detection of Syndiniales sequences, our ability to classify these sequences, and identify what life stage they represent, is still very limited. Dinospores might be expected to represent the greatest number of sequences because they are released in larger numbers, but not all dinospores will eventually infect a host. Our data suggest that the majority of Syndiniales sequences might represent free‐living dinospores. Furthermore, we would expect that the appearance or increase of parasite ASVs at a given time is indicative of the presence of its host(s) as well. As dinospores have not been documented to survive outside of their host for longer than a few weeks, their disappearance would indicate the absence of their host, possibly due to high mortality from infections, as well as dinospore loss. However, several members of Syndiniales (particularly *Amoebophrya*) are known to have multiple hosts (Coats & Park, 2002); thus we cannot assume near‐absolute co‐occurrence (even with a time lag) as we might if there were only single host specificity.

To find putative species–species interactions, we used SparCC, which is intended for sparse compositional data. A recently published database of observationally or experimentally confirmed protist interactions obtained from an extensive literature review found 200 out of 2422 interactions involved parasitism by a member of Syndiniales (Bjorbækmo et al., 2020). Half of these were between the clade II genus *Amoeobphrya* and dinoflagellates, with a few more between *Amoebophrya* and ciliates or acantharians. The rest were between Syndiniales clades I or II and rhizarians, cercozoans, ciliates, chrysophytes, or dinoflagellates. Four Syndiniales species among those characterized in the Bjorbækmo et al. (2020) database, were observed to have more than one host (in three cases, multiple dinoflagellate hosts, and in one case multiple ciliate hosts). *Amoebophrya* spp. have almost exclusively been associated with dinoflagellates with the exception of a few interactions with ciliates and acantharians. Clade I and the remaining members of clade II were only identified to interact with rhizarians. In contrast to comprising one‐tenth of the interactions in the PIDA database, Syndiniales parasites dominated the co‐occurrence‐based associations identified by the TARA oceans study (Lima‐mendez et al., 2015).

In our co‐occurrence analysis, we found approximately one‐sixth of the correlation‐based interactions (that were significant with *r* > 0.6) included a member of Syndiniales. We found correlations between Syndiniales clade I ASVs and Dinophyceae, Telonemia, Cryomonadida, Pyramimonadales, Tintinnids, Gymnodiniales, Bacillariophyta, Raphidophyceae, Oomycota, Strombidiida, Prorocentrales, Choreotrichida, Thecofilosa, MAST‐3, Chlorachnida, prymnesiales, chlorellales and other members of Syndiniales. We found correlations between Syndiniales clade II and dinophyceae, MAST‐7, Bacillariophyta, Mamiellales, MOCH‐2, Gymnodiniales, Cryomonadida, Pelagophyceae, Tintinnidae, Chlorellales and other members of Syndiniales. Similarly, Sassenhagen et al. (2020) found associations between Syndiniales clades I, II and IV and both cercozoans and diatoms. Interactions were identified through co‐occurrence analyses of environmental samples as well as sequences from single‐cell isolates from these samples, and some single‐cell isolates were associated with multiple Syndiniales sequences. The majority of Syndiniales ASVs with significant correlations had correlations with multiple other ASVs, which is consistent with numerous other studies (de Vargas et al., 2015; Sassenhagen et al., 2020).

While Syndiniales Groups I and II had considerably greater ASVs and sequences, there were 39 Group III ASVs and some were present throughout the year; little is known about this group despite its significant presence in marine communities. The ubiquitously present Syndiniales Group III ASV, ASV 14, was correlated with ASV 25 (*Bathycoccus*), ASV 171 (Katablepharidophyta), ASV 40 (Mamiellales) and ASV 133 (Dictyophyceae) (*r* = 0.69, 0,69, 0.68 and 0.62, respectively). To our knowledge, no specific associations involving Syndiniales Group III have been reported elsewhere thus far, but these could be microbes to target to detect potential hosts of Group III Syndinales. However, in our co‐occurrence study, we removed all metazoans and these are also poorly resolved with 18 S rRNA sequences. Utilizing another marker gene sequence such as COXI might be better for detecting parasite–host interactions of Group III if these involve metazoans.

Our effort has yielded a number of potential interactions, which are now important candidates for further study on environmental samples. Some of the taxa implicated as parasite–host pairs from our analysis, such as *Gymnodinium* and *Amoebophrya*, have been previously validated on the genus level (Coats et al., 2010)—but limitations in the length of our amplicon and the current sequence databases do not allow for confident classification at higher taxonomic resolution. We were also able to find two other parasite–host pairs that could be confirmed in other ways: The dinoflagellate *Karlodinium* and its parasite *Amoebophrya*, which we have maintained in culture, and one pair of co‐occurring ASVs that was later clearly observed microscopically during a large dinoflagellate bloom in 2018 (Figure 1A). In both these cases, there was observable co‐occurrence of the ASVs (for the latter pair, these were extrapolated from their abundance in the 2018 bloom sample as well as co‐occurrence in 2016 samples), but neither pair was identified using the SparCC. This is likely due to their transience; both were present in abundance for a very small number of time points. Given the clear seasonality of our site (Figure 3B), a multi‐year time series might allow for more non‐zero time points for a given host that only appears for a small part of the year.

Another crucial confounding factor in the use of sequence‐based time‐series analyses to identify parasitic interactions is the lack of partitioning between parasite life stages. Sequencing environmental samples obtained through a 0.2 μm filter captures both dinospores and active infections. However, due to the difference in size between host dinoflagellates and their dinospores, we hypothesized that size fractionation using different filter sizes may inform the progression of infection. Early infections would be defined by the presence of Syndiniales ASVs in a >2‐ or >10‐μm‐filtered fraction (while inside the host cell) but not in a 0.2‐μm‐filtered fraction, as dinospores would not be released at large quantities. Later on, in the infection cycle, dinospores would be detectable in the smaller size fraction. From a comparison of a 0.2‐ and 10‐μm size fractions, we appear to be able to separate the dinospore sequence signal from that of active infections at the SIO Pier (Figure S2). This opens up new strategies for following the Syndinales population in its life cycle stages, perhaps in conjunction with fluorescence microscopy of filters. The TARA oceans study reported size‐fractionated sequence data and found a large majority of Syndiniales clade I and II sequences to be in the pico‐nano (0.8–5 μm) fraction (de Vargas et al., 2015), so it is likely that most Syndiniales sequences represent dinospores rather than infections. Sassenhagen et al. (2020) also found a greater abundance of Syndiniales sequences in a smaller size fraction, along with fluctuations in the size‐fraction distribution over time. However, one caveat is that there were a few dinoflagellate ASVs that passed through the 10 μm filter, which might represent smaller cells or extracellular DNA. These included ASVs classified within *Gymnodinium*, *Heterocapsa* and *Prorocentrum*. So some potential hosts may not be able to be separated using this method and other combinations of filter sizes may be more useful.

Members of the Syndiniales clearly play an active role in the microbial ecosystem at the SIO pier. In addition to the simplistic single parasite–single host interactions, it is known that some of these might be generalists in terms of their hosts. In a system with hosts vulnerable to multiple parasite species and parasites lethal to multiple host species, there might be a dilution of signal when analysing time series. Additional dynamics, like competition between Syndiniales species for host resources, and even consumption of dinospores by protists, have not been properly accounted for in ecosystem‐level analyses. We suggest that high‐frequency and high‐throughput environmental sequencing has the potential to help track dynamics of parasites and hosts, but only with careful consideration of how to enumerate the cells and distinguish among their life stages.

## AUTHOR CONTRIBUTION

Conceptualization: B.P., M.N. Lab and bioinformatic analyses: M.N. Writing – original draft: M.N. Writing – review and editing: B.P., M.N.

## CONFLICT OF INTEREST

The authors declare that there are no conflicts of interest.

## Supporting information


**APPENDIX S1.** Supporting Information

**TABLE S1.**Full BIOM table

**TABLE S2.**Full SparCC table

## Data Availability

The environmental data used in this study are available in supplementary materials. Sequence data have been deposited in NCBI Genbank under Bioproject PRJNA903390, Accessions: SRR22349159‐SRR22349262.
