# Supplementary material for: Diversity and putative interactions of parasitic alveolates belonging to Syndiniales at a coastal Pacific site
Source: Environ Microbiol Rep. 2023 Feb 13;15(3):157–69. doi: 10.1111/1758-2229.13138 (PMC10464665; doi:10.1111/1758-2229.13138)
Supplement: Supplementary file 1 — APPENDIX S1. Supporting Information [file EMI4-15-157-s001.docx]

SUPPLEMENTARY METHODS AND FIGURES

**Illumina 2-step protocol from RTL Genomics**

Samples were amplified for sequencing in a two-step process. The forward primer was constructed with (5’-3’) the Illumina i5 sequencing primer (TCGTCGGCAGCGTCAGATGTGTATAAGAGACAG) and the Euk_1391F primer (Amaral-Zettler et al., 2009). The reverse primer was constructed with (5’-3’) the Illumina i7 sequencing primer (GTCTCGTGGGCTCGGAGATGTGTATAAGAGACAG) and the EukBr primer (Stoeck et al., 2010). Amplifications were performed in 25 µl reactions with Qiagen HotStar Taq master mix (Qiagen Inc, Valencia, California), 1ul of each 5µM primer, and 1µl of template. Reactions were performed on ABI Veriti thermocyclers (Applied Biosystems, Carlsbad, California) under the following thermal profile: 95^○^C for 5 min, then 35 cycles of 94^○^C for 30 s, 54^○^C for 40 s, 72^○^C for 1 min, followed by one cycle of 72^○^C for 10 min and 4^○^C hold.

Products from the first stage amplification were added to a second PCR based on qualitatively determine concentrations. Primers for the second PCR were designed based on the Illumina Nextera PCR primers as follows: Forward - AATGATACGGCGACCACCGAGATCTACAC[i5index]TCGTCGGCAGCGTC and Reverse - CAAGCAGAAGACGGCATACGAGAT[i7index]GTCTCGTGGGCTCGG. The second stage amplification was run the same as the first stage except for 10 cycles.

Amplification products were visualized with eGels (Life Technologies, Grand Island, New York). Products were then pooled equimolar and each pool was size selected in two rounds using SPRIselect Reagent (Beckman Coulter, Indianapolis, Indiana) in a 0.75 ratio for both rounds. Size selected pools were then quantified using the Qubit 4 Fluorometer (Life Technologies) and loaded on an Illumina MiSeq (Illumina, Inc. San Diego, California) 2x300 flow cell at 10pM.

***Mock communities and replicates***

We sequenced several mock communities along with the seawater samples. Mock communities were created by pipetting known proportions (based on microscopic enumeration; Fig. S1) of cells from different cultures onto a 0.2 µm filter. Cultures used were *Amoebophrya sp., Tetraselmis Sp., Heterocapsa rotundata, Gymnodinium sp., Karlodinium sp., Lingulodinium sp., Prorocentrum sp.;* the latter five are dinoflagellates and might be expected to be overrepresented in the data due to high 18S copy number . Dilutions of 1:10, 1:100, and 1:1000 were also sequenced. Filters were then processed in the same way as the environmental samples, described above. Additionally, we sequenced replicates of certain samples (including the same samples sequenced at RTL Genomics and using our own library prep).

***Filtration experiments***

We sequenced a community from July 2017 onto both a 0.2 (total community) and 10 micron filter (uninfected and infected dinoflagellates). Other filtration experiments on other dates are also listed. We isolated DNA and sequenced and analyzed the sequence data as described.

SUPPLEMENTARY FIGURES

***Mock Communities***

**Fig. S1** (A) 18S sequence results of eukaryotic mock communities. Two technical replicates were sequenced, and replicate 1 was also diluted 10-fold, 100-fold, and 1000-fold. Actual proportions of cells added shown in the bottom row; all other rows represent proportions of sequences found in various replicates. Several of the communities contain contaminants (“other” and “Copepod” sequences). (B**)**18S sequence results of combinations of *Karlodinium*(host) and *Amoebophrya*(Syndiniales parasite) cells. "MC" was a mock community where 700,000 *Karlodinium* cells and 700,000 *Amoebophrya* dinospores were combined and immediately filtered. "Culture" was a *Karlodinium* and *Amoebophrya* co-culture where the abundances of each life stage were enumerated by microscopy prior to filtration (reported in table).


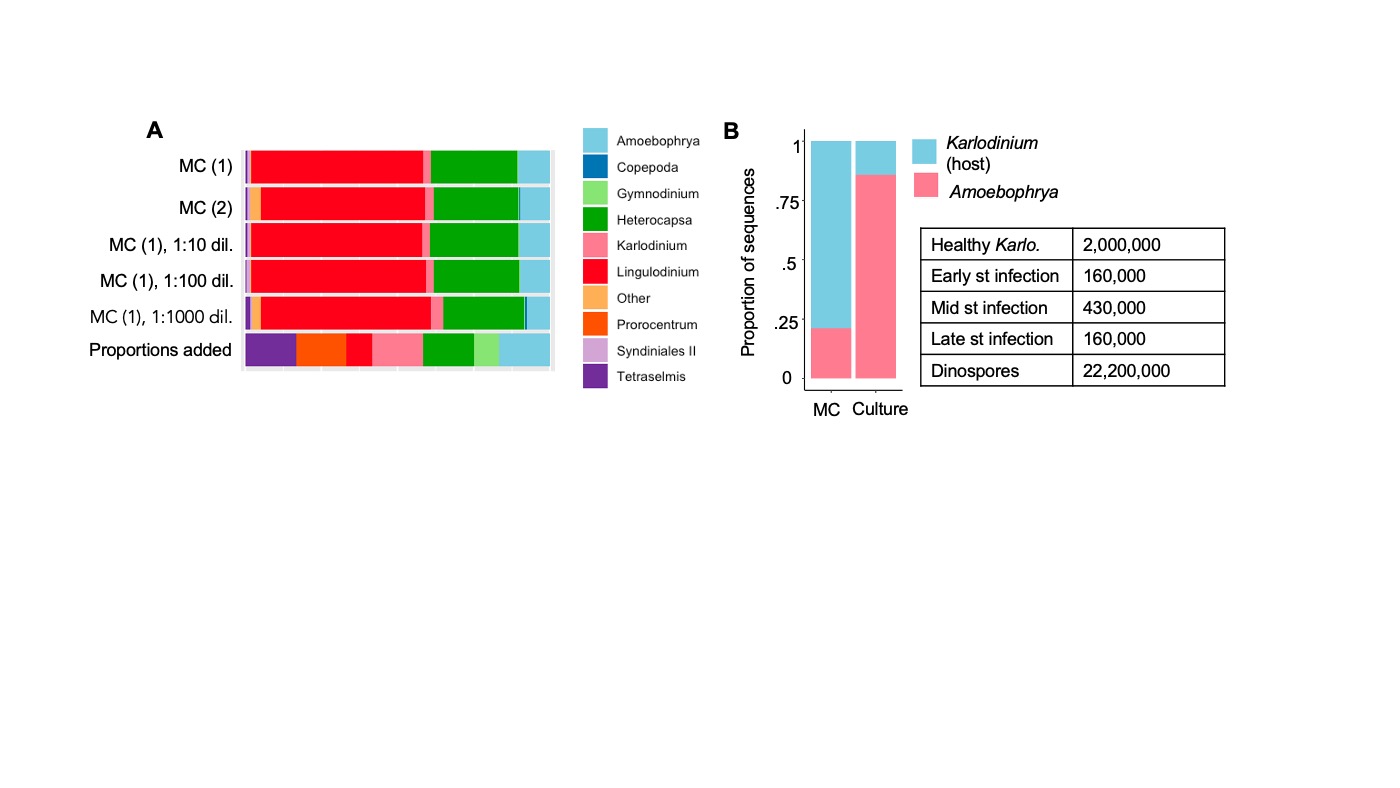


***Filtration experiments***

**Fig. S2**(A) Schematic representation of filtration procedure for different samples shown; filter collected colored grey (B) Relative taxonomic abundances of of all dinoflagellate (left) and all Syndiniales (right) ASVs amongst several different filter size fractions. Numbers designate the filtration method from (A). Letters represent date sampled; A – 10 July 2017; B & C – 17 July 2017; D & E – 27 July 2017.  Asterisks indicate that the sample contained sequences from groups III and IV. (C) Comparison of three Syndiniales ASVs filtered at two size fractions each, one week apart. Measurements are shown as a proportion of total sequences.


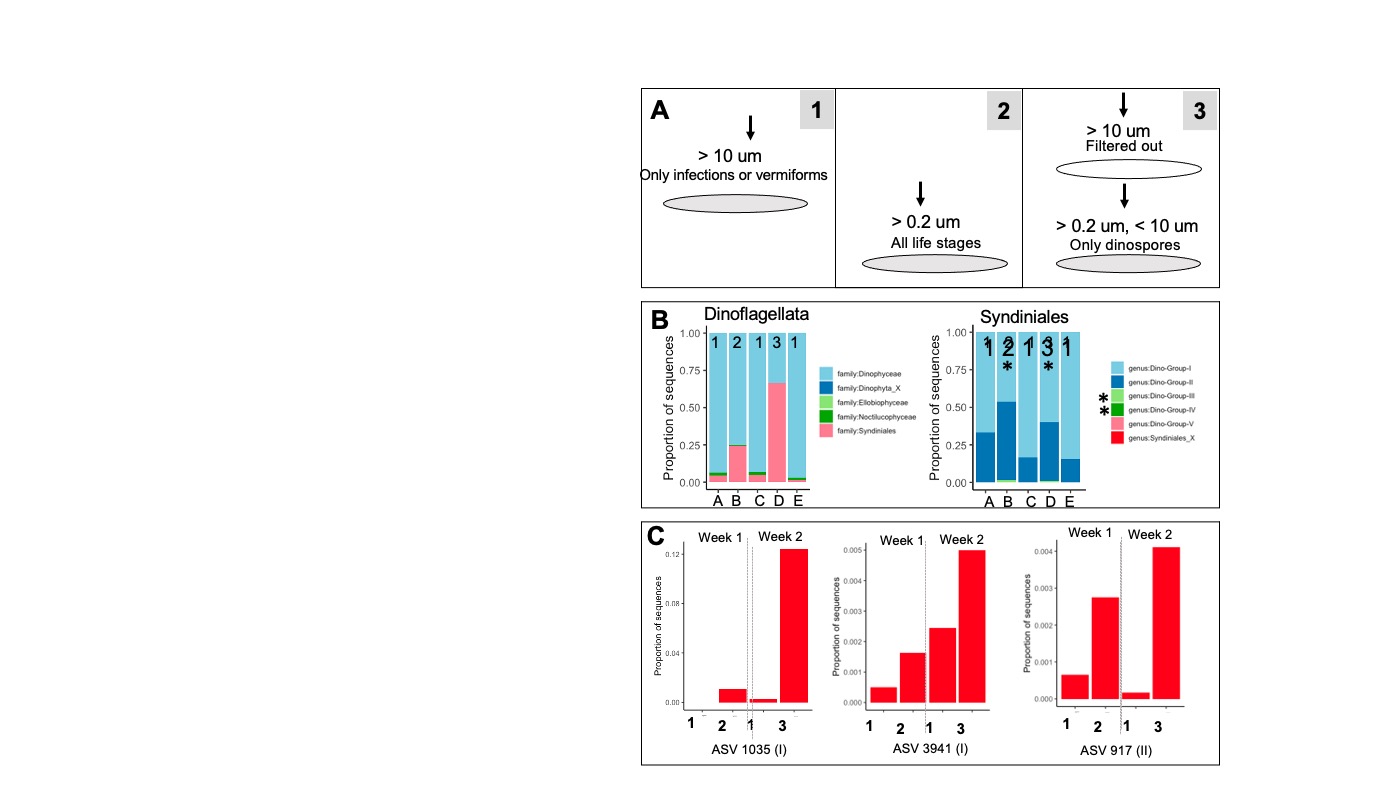


**REFERENCES**

Amaral-Zettler, L. A., McCliment, E. A., Ducklow, H. W., & Huse, S. M. (2009). A method for studying protistan diversity using massively parallel sequencing of V9 hypervariable regions of small-subunit ribosomal RNA Genes. *PLoS ONE*, *4*(7), 1–9. <https://doi.org/10.1371/journal.pone.0006372>

Stoeck, T., Bass, D., Nebel, M., Christen, R., Jones, M. D. M., Breiner, H. W., & Richards, T. A. (2010). Multiple marker parallel tag environmental DNA sequencing reveals a highly complex eukaryotic community in marine anoxic water. *Molecular Ecology*, *19*, 21–31. <https://doi.org/10.1111/j.1365-294X.2009.04480.x>

**Supplementary Table 1**

Full BIOM table, see separate file

**Supplementary Table 2**

Full SparCC table, see separate file
