# Supplementary material for: Diversity and putative interactions of parasitic alveolates belonging to Syndiniales at a coastal Pacific site
Source: Environ Microbiol Rep. 2023 Feb 13;15(3):157–69. doi: 10.1111/1758-2229.13138 (PMC10464665; doi:10.1111/1758-2229.13138)
Supplement: Supplementary file 3 — TABLE S2.Full SparCC table [file EMI4-15-157-s003.docx]

| **Var1** | Tax4_x | Tax5_x | **Var2** | Tax4_y | Tax5_y | **Correlation** | p |
| --- | --- | --- | --- | --- | --- | --- | --- |
| **ASV_4** | Cryptophyceae_X | Cryptomonadales | **ASV_20** | Fungi_XX | Fungi_XXX | **0.93** | 0 |
| **ASV_25** | Mamiellales | Bathycoccaceae | **ASV_40** | Mamiellales | Mamiellaceae | **0.91** | 0 |
| **ASV_3** | Bacillariophyta_X | Polar-centric-Mediophyceae | **ASV_35** | Chlorellales | Chlorellales_X | **0.87** | 0 |
| **ASV_900** | Pyramimonadales_X | Pyramimonadales_XX | **ASV_1014** | Gymnodiniales | Gymnodiniaceae | **0.82** | 0 |
| **ASV_3** | Bacillariophyta_X | Polar-centric-Mediophyceae | **ASV_26** | Bacillariophyta_X | Polar-centric-Mediophyceae | **0.81** | 0 |
| **ASV_3** | Bacillariophyta_X | Polar-centric-Mediophyceae | **ASV_28** | Bacillariophyta_X | Polar-centric-Mediophyceae | **0.81** | 0 |
| **ASV_26** | Bacillariophyta_X | Polar-centric-Mediophyceae | **ASV_35** | Chlorellales | Chlorellales_X | **0.80** | 0 |
| **ASV_28** | Bacillariophyta_X | Polar-centric-Mediophyceae | **ASV_35** | Chlorellales | Chlorellales_X | **0.80** | 0 |
| **ASV_779** | Raphidophyceae_X | Raphidophyceae_XX | **ASV_842** | Dino-Group-I | Dino-Group-I-Clade-5 | **0.78** | 0 |
| **ASV_2** | Bacillariophyta_X | Polar-centric-Mediophyceae | **ASV_3** | Bacillariophyta_X | Polar-centric-Mediophyceae | **0.78** | 0 |
| **ASV_2** | Bacillariophyta_X | Polar-centric-Mediophyceae | **ASV_36** | Bacillariophyta_X | Polar-centric-Mediophyceae | **0.77** | 0 |
| **ASV_3** | Bacillariophyta_X | Polar-centric-Mediophyceae | **ASV_1711** | Cryomonadida | Protaspa-lineage | **0.77** | 0 |
| **ASV_15** | Mamiellales | Bathycoccaceae | **ASV_25** | Mamiellales | Bathycoccaceae | **0.77** | 0 |
| **ASV_779** | Raphidophyceae_X | Raphidophyceae_XX | **ASV_1014** | Gymnodiniales | Gymnodiniaceae | **0.77** | 0 |
| **ASV_28** | Bacillariophyta_X | Polar-centric-Mediophyceae | **ASV_58** | Pelagomonadales | Pelagomonadaceae | **0.77** | 0 |
| **ASV_25** | Mamiellales | Bathycoccaceae | **ASV_44** | Mamiellales | Mamiellaceae | **0.77** | 0 |
| **ASV_779** | Raphidophyceae_X | Raphidophyceae_XX | **ASV_933** | Tintinnida | Eutintinnidae | **0.77** | 0 |
| **ASV_2** | Bacillariophyta_X | Polar-centric-Mediophyceae | **ASV_58** | Pelagomonadales | Pelagomonadaceae | **0.77** | 0 |
| **ASV_40** | Mamiellales | Mamiellaceae | **ASV_44** | Mamiellales | Mamiellaceae | **0.76** | 0 |
| **ASV_607** | Dinophyceae_X | Dinophyceae_XX | **ASV_779** | Raphidophyceae_X | Raphidophyceae_XX | **0.76** | 0 |
| **ASV_2** | Bacillariophyta_X | Polar-centric-Mediophyceae | **ASV_26** | Bacillariophyta_X | Polar-centric-Mediophyceae | **0.76** | 0 |
| **ASV_40** | Mamiellales | Mamiellaceae | **ASV_68** | Mamiellales | Mamiellaceae | **0.76** | 0 |
| **ASV_779** | Raphidophyceae_X | Raphidophyceae_XX | **ASV_951** | Telonemia_XX | Telonemia-Group-2 | **0.76** | 0 |
| **ASV_35** | Chlorellales | Chlorellales_X | **ASV_281** | Bacillariophyta_X | Raphid-pennate | **0.75** | 0 |
| **ASV_951** | Telonemia_XX | Telonemia-Group-2 | **ASV_1054** | Telonemia_XX | Telonemia-Group-2 | **0.75** | 0 |
| **ASV_963** | MAST-3 | MAST-3B | **ASV_983** | Chlorellales | Chlorellales_X | **0.75** | 0 |
| **ASV_36** | Bacillariophyta_X | Polar-centric-Mediophyceae | **ASV_58** | Pelagomonadales | Pelagomonadaceae | **0.75** | 0 |
| **ASV_28** | Bacillariophyta_X | Polar-centric-Mediophyceae | **ASV_1711** | Cryomonadida | Protaspa-lineage | **0.75** | 0 |
| **ASV_607** | Dinophyceae_X | Dinophyceae_XX | **ASV_842** | Dino-Group-I | Dino-Group-I-Clade-5 | **0.75** | 0 |
| **ASV_900** | Pyramimonadales_X | Pyramimonadales_XX | **ASV_967** | Choreotrichida | Lynnellidae | **0.75** | 0 |
| **ASV_586** | Dino-Group-II | Dino-Group-II_X | **ASV_944** | Dinophyceae_X | Dinophyceae_XX | **0.74** | 0 |
| **ASV_35** | Chlorellales | Chlorellales_X | **ASV_1711** | Cryomonadida | Protaspa-lineage | **0.74** | 0 |
| **ASV_2** | Bacillariophyta_X | Polar-centric-Mediophyceae | **ASV_28** | Bacillariophyta_X | Polar-centric-Mediophyceae | **0.74** | 0 |
| **ASV_40** | Mamiellales | Mamiellaceae | **ASV_74** | Mamiellales | Mamiellaceae | **0.74** | 0 |
| **ASV_26** | Bacillariophyta_X | Polar-centric-Mediophyceae | **ASV_28** | Bacillariophyta_X | Polar-centric-Mediophyceae | **0.74** | 0 |
| **ASV_967** | Choreotrichida | Lynnellidae | **ASV_1014** | Gymnodiniales | Gymnodiniaceae | **0.74** | 0 |
| **ASV_3** | Bacillariophyta_X | Polar-centric-Mediophyceae | **ASV_36** | Bacillariophyta_X | Polar-centric-Mediophyceae | **0.74** | 0 |
| **ASV_900** | Pyramimonadales_X | Pyramimonadales_XX | **ASV_950** | Syndiniales_X | Syndiniales_XX | **0.74** | 0 |
| **ASV_108** | Dino-Group-II | Dino-Group-II-Clade-6 | **ASV_202** | MAST-7 | MAST-7B | **0.74** | 0 |
| **ASV_842** | Dino-Group-I | Dino-Group-I-Clade-5 | **ASV_951** | Telonemia_XX | Telonemia-Group-2 | **0.73** | 0 |
| **ASV_15** | Mamiellales | Bathycoccaceae | **ASV_93** | Katablepharidales | Katablepharidales_X | **0.73** | 0 |
| **ASV_4** | Cryptophyceae_X | Cryptomonadales | **ASV_7** | Cryptophyceae_X | Cryptomonadales | **0.73** | 0 |
| **ASV_15** | Mamiellales | Bathycoccaceae | **ASV_28** | Bacillariophyta_X | Polar-centric-Mediophyceae | **0.73** | 0 |
| **ASV_2** | Bacillariophyta_X | Polar-centric-Mediophyceae | **ASV_35** | Chlorellales | Chlorellales_X | **0.73** | 0 |
| **ASV_25** | Mamiellales | Bathycoccaceae | **ASV_68** | Mamiellales | Mamiellaceae | **0.73** | 0 |
| **ASV_961** | Dinophyceae_X | Dinophyceae_XX | **ASV_1014** | Gymnodiniales | Gymnodiniaceae | **0.73** | 0 |
| **ASV_28** | Bacillariophyta_X | Polar-centric-Mediophyceae | **ASV_237** | Dino-Group-II | Dino-Group-II-Clade-10-and-11 | **0.73** | 0 |
| **ASV_779** | Raphidophyceae_X | Raphidophyceae_XX | **ASV_1037** | Dinophyceae_X | Dinophyceae_XX | **0.73** | 0 |
| **ASV_28** | Bacillariophyta_X | Polar-centric-Mediophyceae | **ASV_150** | Cryomonadida | Cryothecomonas-lineage | **0.73** | 0 |
| **ASV_3** | Bacillariophyta_X | Polar-centric-Mediophyceae | **ASV_281** | Bacillariophyta_X | Raphid-pennate | **0.73** | 0 |
| **ASV_276** | Prymnesiales | Prymnesiophyceae_Clade_B4 | **ASV_629** | Prymnesiales | Prymnesiophyceae_Clade_B5 | **0.73** | 0 |
| **ASV_15** | Mamiellales | Bathycoccaceae | **ASV_40** | Mamiellales | Mamiellaceae | **0.72** | 0 |
| **ASV_28** | Bacillariophyta_X | Polar-centric-Mediophyceae | **ASV_482** | Chlorellales | Chlorellales_X | **0.72** | 0 |
| **ASV_779** | Raphidophyceae_X | Raphidophyceae_XX | **ASV_961** | Dinophyceae_X | Dinophyceae_XX | **0.72** | 0 |
| **ASV_26** | Bacillariophyta_X | Polar-centric-Mediophyceae | **ASV_36** | Bacillariophyta_X | Polar-centric-Mediophyceae | **0.72** | 0 |
| **ASV_842** | Dino-Group-I | Dino-Group-I-Clade-5 | **ASV_961** | Dinophyceae_X | Dinophyceae_XX | **0.72** | 0 |
| **ASV_900** | Pyramimonadales_X | Pyramimonadales_XX | **ASV_961** | Dinophyceae_X | Dinophyceae_XX | **0.72** | 0 |
| **ASV_2** | Bacillariophyta_X | Polar-centric-Mediophyceae | **ASV_150** | Cryomonadida | Cryothecomonas-lineage | **0.72** | 0 |
| **ASV_3** | Bacillariophyta_X | Polar-centric-Mediophyceae | **ASV_58** | Pelagomonadales | Pelagomonadaceae | **0.72** | 0 |
| **ASV_842** | Dino-Group-I | Dino-Group-I-Clade-5 | **ASV_950** | Syndiniales_X | Syndiniales_XX | **0.72** | 0 |
| **ASV_842** | Dino-Group-I | Dino-Group-I-Clade-5 | **ASV_1037** | Dinophyceae_X | Dinophyceae_XX | **0.71** | 0 |
| **ASV_67** | Bacillariophyta_X | Polar-centric-Mediophyceae | **ASV_1014** | Gymnodiniales | Gymnodiniaceae | **0.71** | 0 |
| **ASV_951** | Telonemia_XX | Telonemia-Group-2 | **ASV_1037** | Dinophyceae_X | Dinophyceae_XX | **0.71** | 0 |
| **ASV_25** | Mamiellales | Bathycoccaceae | **ASV_202** | MAST-7 | MAST-7B | **0.71** | 0 |
| **ASV_1711** | Cryomonadida | Protaspa-lineage | **ASV_2204** | Dino-Group-I | Dino-Group-I-Clade-1 | **0.71** | 0 |
| **ASV_25** | Mamiellales | Bathycoccaceae | **ASV_74** | Mamiellales | Mamiellaceae | **0.71** | 0 |
| **ASV_961** | Dinophyceae_X | Dinophyceae_XX | **ASV_1037** | Dinophyceae_X | Dinophyceae_XX | **0.71** | 0 |
| **ASV_779** | Raphidophyceae_X | Raphidophyceae_XX | **ASV_900** | Pyramimonadales_X | Pyramimonadales_XX | **0.71** | 0 |
| **ASV_119** | Dinophyceae_X | Dinophyceae_XX | **ASV_164** | Strombidiida | Strombidiidae_K | **0.71** | 0 |
| **ASV_25** | Mamiellales | Bathycoccaceae | **ASV_108** | Dino-Group-II | Dino-Group-II-Clade-6 | **0.71** | 0 |
| **ASV_912** | MOCH-2 | MOCH-2_X | **ASV_958** | Dino-Group-II | Dino-Group-II-Clade-10-and-11 | **0.71** | 0 |
| **ASV_44** | Mamiellales | Mamiellaceae | **ASV_68** | Mamiellales | Mamiellaceae | **0.71** | 0 |
| **ASV_93** | Katablepharidales | Katablepharidales_X | **ASV_150** | Cryomonadida | Cryothecomonas-lineage | **0.71** | 0 |
| **ASV_842** | Dino-Group-I | Dino-Group-I-Clade-5 | **ASV_900** | Pyramimonadales_X | Pyramimonadales_XX | **0.71** | 0 |
| **ASV_1062** | Bacillariophyta_X | Radial-centric-basal-Coscinodiscophyceae | **ASV_1224** | Bacillariophyta_X | Araphid-pennate | **0.71** | 0 |
| **ASV_908** | Gymnodiniales | Gymnodiniaceae | **ASV_1057** | Dino-Group-II | Dino-Group-II-Clade-32 | **0.71** | 0 |
| **ASV_961** | Dinophyceae_X | Dinophyceae_XX | **ASV_967** | Choreotrichida | Lynnellidae | **0.71** | 0 |
| **ASV_36** | Bacillariophyta_X | Polar-centric-Mediophyceae | **ASV_150** | Cryomonadida | Cryothecomonas-lineage | **0.71** | 0 |
| **ASV_67** | Bacillariophyta_X | Polar-centric-Mediophyceae | **ASV_779** | Raphidophyceae_X | Raphidophyceae_XX | **0.71** | 0 |
| **ASV_28** | Bacillariophyta_X | Polar-centric-Mediophyceae | **ASV_328** | Dino-Group-II | Dino-Group-II-Clade-21 | **0.71** | 0 |
| **ASV_779** | Raphidophyceae_X | Raphidophyceae_XX | **ASV_950** | Syndiniales_X | Syndiniales_XX | **0.71** | 0 |
| **ASV_772** | Chlorarachnida | Chlorarachnida_X | **ASV_983** | Chlorellales | Chlorellales_X | **0.70** | 0 |
| **ASV_607** | Dinophyceae_X | Dinophyceae_XX | **ASV_1037** | Dinophyceae_X | Dinophyceae_XX | **0.70** | 0 |
| **ASV_950** | Syndiniales_X | Syndiniales_XX | **ASV_1037** | Dinophyceae_X | Dinophyceae_XX | **0.70** | 0 |
| **ASV_1037** | Dinophyceae_X | Dinophyceae_XX | **ASV_2086** | Strombidiida | Strombidiidae_Q | **0.70** | 0 |
| **ASV_779** | Raphidophyceae_X | Raphidophyceae_XX | **ASV_1077** | Oomycota_X | Oomycota_XX | **0.70** | 0 |
| **ASV_1014** | Gymnodiniales | Gymnodiniaceae | **ASV_1037** | Dinophyceae_X | Dinophyceae_XX | **0.70** | 0 |
| **ASV_26** | Bacillariophyta_X | Polar-centric-Mediophyceae | **ASV_1711** | Cryomonadida | Protaspa-lineage | **0.70** | 0 |
| **ASV_3** | Bacillariophyta_X | Polar-centric-Mediophyceae | **ASV_237** | Dino-Group-II | Dino-Group-II-Clade-10-and-11 | **0.70** | 0 |
| **ASV_36** | Bacillariophyta_X | Polar-centric-Mediophyceae | **ASV_294** | Peridiniales | Amphidiniopsidaceae | **0.70** | 0 |
| **ASV_12** | Cryptophyceae_X | Cryptomonadales | **ASV_255** | Cryptophyceae_X | Cryptomonadales | **0.70** | 0 |
| **ASV_842** | Dino-Group-I | Dino-Group-I-Clade-5 | **ASV_933** | Tintinnida | Eutintinnidae | **0.70** | 0 |
| **ASV_967** | Choreotrichida | Lynnellidae | **ASV_1259** | Dinophyceae_X | Dinophyceae_XX | **0.70** | 0 |
| **ASV_12** | Cryptophyceae_X | Cryptomonadales | **ASV_27** | Cryptophyceae_X | Cryptomonadales | **0.69** | 0 |
| **ASV_14** | Dino-Group-III | Dino-Group-III_X | **ASV_25** | Mamiellales | Bathycoccaceae | **0.69** | 0 |
| **ASV_28** | Bacillariophyta_X | Polar-centric-Mediophyceae | **ASV_93** | Katablepharidales | Katablepharidales_X | **0.69** | 0 |
| **ASV_950** | Syndiniales_X | Syndiniales_XX | **ASV_951** | Telonemia_XX | Telonemia-Group-2 | **0.69** | 0 |
| **ASV_842** | Dino-Group-I | Dino-Group-I-Clade-5 | **ASV_1014** | Gymnodiniales | Gymnodiniaceae | **0.69** | 0 |
| **ASV_900** | Pyramimonadales_X | Pyramimonadales_XX | **ASV_1037** | Dinophyceae_X | Dinophyceae_XX | **0.69** | 0 |
| **ASV_328** | Dino-Group-II | Dino-Group-II-Clade-21 | **ASV_1711** | Cryomonadida | Protaspa-lineage | **0.69** | 0 |
| **ASV_950** | Syndiniales_X | Syndiniales_XX | **ASV_1014** | Gymnodiniales | Gymnodiniaceae | **0.69** | 0 |
| **ASV_14** | Dino-Group-III | Dino-Group-III_X | **ASV_171** | Katablepharidales | Katablepharidales_X | **0.69** | 0 |
| **ASV_1014** | Gymnodiniales | Gymnodiniaceae | **ASV_1062** | Bacillariophyta_X | Radial-centric-basal-Coscinodiscophyceae | **0.69** | 0 |
| **ASV_28** | Bacillariophyta_X | Polar-centric-Mediophyceae | **ASV_137** | Bacillariophyta_X | Raphid-pennate | **0.69** | 0 |
| **ASV_237** | Dino-Group-II | Dino-Group-II-Clade-10-and-11 | **ASV_1711** | Cryomonadida | Protaspa-lineage | **0.69** | 0 |
| **ASV_28** | Bacillariophyta_X | Polar-centric-Mediophyceae | **ASV_46** | Strombidiida | Strombidiidae_M | **0.69** | 0 |
| **ASV_36** | Bacillariophyta_X | Polar-centric-Mediophyceae | **ASV_625** | Dino-Group-II | Dino-Group-II-Clade-10-and-11 | **0.69** | 0 |
| **ASV_607** | Dinophyceae_X | Dinophyceae_XX | **ASV_933** | Tintinnida | Eutintinnidae | **0.69** | 0 |
| **ASV_607** | Dinophyceae_X | Dinophyceae_XX | **ASV_951** | Telonemia_XX | Telonemia-Group-2 | **0.69** | 0 |
| **ASV_779** | Raphidophyceae_X | Raphidophyceae_XX | **ASV_1057** | Dino-Group-II | Dino-Group-II-Clade-32 | **0.69** | 0 |
| **ASV_629** | Prymnesiales | Prymnesiophyceae_Clade_B5 | **ASV_944** | Dinophyceae_X | Dinophyceae_XX | **0.69** | 0 |
| **ASV_779** | Raphidophyceae_X | Raphidophyceae_XX | **ASV_1054** | Telonemia_XX | Telonemia-Group-2 | **0.69** | 0 |
| **ASV_950** | Syndiniales_X | Syndiniales_XX | **ASV_2086** | Strombidiida | Strombidiidae_Q | **0.69** | 0 |
| **ASV_28** | Bacillariophyta_X | Polar-centric-Mediophyceae | **ASV_2160** | Dino-Group-I | Dino-Group-I-Clade-4 | **0.68** | 0 |
| **ASV_951** | Telonemia_XX | Telonemia-Group-2 | **ASV_1014** | Gymnodiniales | Gymnodiniaceae | **0.68** | 0 |
| **ASV_232** | Pelagophyceae_X | Pelagophyceae_XX | **ASV_586** | Dino-Group-II | Dino-Group-II_X | **0.68** | 0 |
| **ASV_951** | Telonemia_XX | Telonemia-Group-2 | **ASV_967** | Choreotrichida | Lynnellidae | **0.68** | 0 |
| **ASV_15** | Mamiellales | Bathycoccaceae | **ASV_150** | Cryomonadida | Cryothecomonas-lineage | **0.68** | 0 |
| **ASV_950** | Syndiniales_X | Syndiniales_XX | **ASV_967** | Choreotrichida | Lynnellidae | **0.68** | 0 |
| **ASV_44** | Mamiellales | Mamiellaceae | **ASV_74** | Mamiellales | Mamiellaceae | **0.68** | 0 |
| **ASV_14** | Dino-Group-III | Dino-Group-III_X | **ASV_40** | Mamiellales | Mamiellaceae | **0.68** | 0 |
| **ASV_933** | Tintinnida | Eutintinnidae | **ASV_950** | Syndiniales_X | Syndiniales_XX | **0.68** | 0 |
| **ASV_779** | Raphidophyceae_X | Raphidophyceae_XX | **ASV_964** | Dino-Group-I | Dino-Group-I-Clade-1 | **0.68** | 0 |
| **ASV_7** | Cryptophyceae_X | Cryptomonadales | **ASV_20** | Fungi_XX | Fungi_XXX | **0.68** | 0 |
| **ASV_22** | Pelagomonadales | Pelagomonadaceae | **ASV_55** | MOCH-2 | MOCH-2_X | **0.68** | 0 |
| **ASV_950** | Syndiniales_X | Syndiniales_XX | **ASV_961** | Dinophyceae_X | Dinophyceae_XX | **0.68** | 0 |
| **ASV_951** | Telonemia_XX | Telonemia-Group-2 | **ASV_961** | Dinophyceae_X | Dinophyceae_XX | **0.68** | 0 |
| **ASV_11** | Dinophyceae_X | Dinophyceae_XX | **ASV_119** | Dinophyceae_X | Dinophyceae_XX | **0.68** | 0 |
| **ASV_31** | Choreotrichida | Leegaardiellidae_B | **ASV_46** | Strombidiida | Strombidiidae_M | **0.68** | 0 |
| **ASV_1679** | Pelagomonadales | Pelagomonadaceae | **ASV_2044** | Collodaria | Collodaria_X | **0.68** | 0 |
| **ASV_95** | Prymnesiales | Prymnesiaceae | **ASV_321** | Prymnesiales | Prymnesiaceae | **0.67** | 0 |
| **ASV_35** | Chlorellales | Chlorellales_X | **ASV_328** | Dino-Group-II | Dino-Group-II-Clade-21 | **0.67** | 0 |
| **ASV_900** | Pyramimonadales_X | Pyramimonadales_XX | **ASV_951** | Telonemia_XX | Telonemia-Group-2 | **0.67** | 0 |
| **ASV_4** | Cryptophyceae_X | Cryptomonadales | **ASV_34** | Pyramimonadales_X | Pyramimonadales_XX | **0.67** | 0 |
| **ASV_28** | Bacillariophyta_X | Polar-centric-Mediophyceae | **ASV_559** | Cryptophyceae_X | Cryptomonadales | **0.67** | 0 |
| **ASV_137** | Bacillariophyta_X | Raphid-pennate | **ASV_482** | Chlorellales | Chlorellales_X | **0.67** | 0 |
| **ASV_607** | Dinophyceae_X | Dinophyceae_XX | **ASV_950** | Syndiniales_X | Syndiniales_XX | **0.67** | 0 |
| **ASV_35** | Chlorellales | Chlorellales_X | **ASV_182** | Dino-Group-II | Dino-Group-II-Clade-44 | **0.67** | 0 |
| **ASV_67** | Bacillariophyta_X | Polar-centric-Mediophyceae | **ASV_900** | Pyramimonadales_X | Pyramimonadales_XX | **0.67** | 0 |
| **ASV_842** | Dino-Group-I | Dino-Group-I-Clade-5 | **ASV_1077** | Oomycota_X | Oomycota_XX | **0.67** | 0 |
| **ASV_708** | Filosa-Thecofilosea_X | Mataza-lineage | **ASV_1711** | Cryomonadida | Protaspa-lineage | **0.67** | 0 |
| **ASV_67** | Bacillariophyta_X | Polar-centric-Mediophyceae | **ASV_607** | Dinophyceae_X | Dinophyceae_XX | **0.67** | 0 |
| **ASV_3** | Bacillariophyta_X | Polar-centric-Mediophyceae | **ASV_294** | Peridiniales | Amphidiniopsidaceae | **0.67** | 0 |
| **ASV_779** | Raphidophyceae_X | Raphidophyceae_XX | **ASV_937** | MAST-1 | MAST-1C | **0.67** | 0 |
| **ASV_586** | Dino-Group-II | Dino-Group-II_X | **ASV_629** | Prymnesiales | Prymnesiophyceae_Clade_B5 | **0.67** | 0 |
| **ASV_929** | Dino-Group-II | Dino-Group-II-Clade-3 | **ASV_942** | Dino-Group-II | Dino-Group-II-Clade-10-and-11 | **0.67** | 0 |
| **ASV_3** | Bacillariophyta_X | Polar-centric-Mediophyceae | **ASV_150** | Cryomonadida | Cryothecomonas-lineage | **0.67** | 0 |
| **ASV_967** | Choreotrichida | Lynnellidae | **ASV_1224** | Bacillariophyta_X | Araphid-pennate | **0.67** | 0 |
| **ASV_1062** | Bacillariophyta_X | Radial-centric-basal-Coscinodiscophyceae | **ASV_1076** | Bacillariophyta_X | Radial-centric-basal-Coscinodiscophyceae | **0.67** | 0 |
| **ASV_933** | Tintinnida | Eutintinnidae | **ASV_1057** | Dino-Group-II | Dino-Group-II-Clade-32 | **0.67** | 0 |
| **ASV_237** | Dino-Group-II | Dino-Group-II-Clade-10-and-11 | **ASV_2204** | Dino-Group-I | Dino-Group-I-Clade-1 | **0.67** | 0 |
| **ASV_877** | Crustacea | Maxillopoda | **ASV_1014** | Gymnodiniales | Gymnodiniaceae | **0.67** | 0 |
| **ASV_28** | Bacillariophyta_X | Polar-centric-Mediophyceae | **ASV_281** | Bacillariophyta_X | Raphid-pennate | **0.67** | 0 |
| **ASV_35** | Chlorellales | Chlorellales_X | **ASV_58** | Pelagomonadales | Pelagomonadaceae | **0.67** | 0 |
| **ASV_281** | Bacillariophyta_X | Raphid-pennate | **ASV_1711** | Cryomonadida | Protaspa-lineage | **0.67** | 0 |
| **ASV_961** | Dinophyceae_X | Dinophyceae_XX | **ASV_1259** | Dinophyceae_X | Dinophyceae_XX | **0.67** | 0 |
| **ASV_779** | Raphidophyceae_X | Raphidophyceae_XX | **ASV_986** | Prorocentrales | Prorocentraceae | **0.66** | 0 |
| **ASV_1570** | Dinophyceae_X | Dinophyceae_XX | **ASV_2160** | Dino-Group-I | Dino-Group-I-Clade-4 | **0.66** | 0 |
| **ASV_15** | Mamiellales | Bathycoccaceae | **ASV_46** | Strombidiida | Strombidiidae_M | **0.66** | 0 |
| **ASV_26** | Bacillariophyta_X | Polar-centric-Mediophyceae | **ASV_58** | Pelagomonadales | Pelagomonadaceae | **0.66** | 0 |
| **ASV_291** | Dino-Group-II | Dino-Group-II-Clade-10-and-11 | **ASV_482** | Chlorellales | Chlorellales_X | **0.66** | 0 |
| **ASV_58** | Pelagomonadales | Pelagomonadaceae | **ASV_1711** | Cryomonadida | Protaspa-lineage | **0.66** | 0 |
| **ASV_2** | Bacillariophyta_X | Polar-centric-Mediophyceae | **ASV_15** | Mamiellales | Bathycoccaceae | **0.66** | 0 |
| **ASV_1037** | Dinophyceae_X | Dinophyceae_XX | **ASV_1054** | Telonemia_XX | Telonemia-Group-2 | **0.66** | 0 |
| **ASV_28** | Bacillariophyta_X | Polar-centric-Mediophyceae | **ASV_36** | Bacillariophyta_X | Polar-centric-Mediophyceae | **0.66** | 0 |
| **ASV_779** | Raphidophyceae_X | Raphidophyceae_XX | **ASV_967** | Choreotrichida | Lynnellidae | **0.66** | 0 |
| **ASV_924** | Pyramimonadales_X | Pyramimonadales_XX | **ASV_986** | Prorocentrales | Prorocentraceae | **0.66** | 0 |
| **ASV_772** | Chlorarachnida | Chlorarachnida_X | **ASV_963** | MAST-3 | MAST-3B | **0.66** | 0 |
| **ASV_1009** | Bacillariophyta_X | Raphid-pennate | **ASV_1223** | Gonyaulacales | Gonyaulacaceae | **0.66** | 0 |
| **ASV_28** | Bacillariophyta_X | Polar-centric-Mediophyceae | **ASV_1570** | Dinophyceae_X | Dinophyceae_XX | **0.66** | 0 |
| **ASV_967** | Choreotrichida | Lynnellidae | **ASV_1009** | Bacillariophyta_X | Raphid-pennate | **0.66** | 0 |
| **ASV_950** | Syndiniales_X | Syndiniales_XX | **ASV_1054** | Telonemia_XX | Telonemia-Group-2 | **0.66** | 0 |
| **ASV_973** | Dino-Group-II | Dino-Group-II-Clade-4 | **ASV_2044** | Collodaria | Collodaria_X | **0.66** | 0 |
| **ASV_924** | Pyramimonadales_X | Pyramimonadales_XX | **ASV_929** | Dino-Group-II | Dino-Group-II-Clade-3 | **0.66** | 0 |
| **ASV_25** | Mamiellales | Bathycoccaceae | **ASV_218** | MAST-4 | MAST-4D | **0.66** | 0 |
| **ASV_772** | Chlorarachnida | Chlorarachnida_X | **ASV_912** | MOCH-2 | MOCH-2_X | **0.66** | 0 |
| **ASV_842** | Dino-Group-I | Dino-Group-I-Clade-5 | **ASV_2086** | Strombidiida | Strombidiidae_Q | **0.66** | 0 |
| **ASV_15** | Mamiellales | Bathycoccaceae | **ASV_58** | Pelagomonadales | Pelagomonadaceae | **0.66** | 0 |
| **ASV_933** | Tintinnida | Eutintinnidae | **ASV_2086** | Strombidiida | Strombidiidae_Q | **0.66** | 0 |
| **ASV_1014** | Gymnodiniales | Gymnodiniaceae | **ASV_1076** | Bacillariophyta_X | Radial-centric-basal-Coscinodiscophyceae | **0.65** | 0 |
| **ASV_779** | Raphidophyceae_X | Raphidophyceae_XX | **ASV_848** | Strombidiida | Strombidiidae | **0.65** | 0 |
| **ASV_586** | Dino-Group-II | Dino-Group-II_X | **ASV_866** | Chlamydomonadales | Chlamydomonadales_X | **0.65** | 0 |
| **ASV_28** | Bacillariophyta_X | Polar-centric-Mediophyceae | **ASV_182** | Dino-Group-II | Dino-Group-II-Clade-44 | **0.65** | 0 |
| **ASV_35** | Chlorellales | Chlorellales_X | **ASV_237** | Dino-Group-II | Dino-Group-II-Clade-10-and-11 | **0.65** | 0 |
| **ASV_961** | Dinophyceae_X | Dinophyceae_XX | **ASV_1062** | Bacillariophyta_X | Radial-centric-basal-Coscinodiscophyceae | **0.65** | 0 |
| **ASV_58** | Pelagomonadales | Pelagomonadaceae | **ASV_625** | Dino-Group-II | Dino-Group-II-Clade-10-and-11 | **0.65** | 0 |
| **ASV_294** | Peridiniales | Amphidiniopsidaceae | **ASV_625** | Dino-Group-II | Dino-Group-II-Clade-10-and-11 | **0.65** | 0 |
| **ASV_937** | MAST-1 | MAST-1C | **ASV_1223** | Gonyaulacales | Gonyaulacaceae | **0.65** | 0 |
| **ASV_560** | Katablepharidales | Katablepharidales_X | **ASV_929** | Dino-Group-II | Dino-Group-II-Clade-3 | **0.65** | 0 |
| **ASV_1013** | Dino-Group-II | Dino-Group-II-Clade-7 | **ASV_1014** | Gymnodiniales | Gymnodiniaceae | **0.65** | 0 |
| **ASV_2** | Bacillariophyta_X | Polar-centric-Mediophyceae | **ASV_37** | Filosa-Thecofilosea_X | Mataza-lineage | **0.65** | 0 |
| **ASV_67** | Bacillariophyta_X | Polar-centric-Mediophyceae | **ASV_203** | Filosa-Imbricatea_X | Novel-clade-2 | **0.65** | 0 |
| **ASV_893** | Gonyaulacales | Goniodomataceae | **ASV_967** | Choreotrichida | Lynnellidae | **0.65** | 0 |
| **ASV_3** | Bacillariophyta_X | Polar-centric-Mediophyceae | **ASV_328** | Dino-Group-II | Dino-Group-II-Clade-21 | **0.65** | 0 |
| **ASV_35** | Chlorellales | Chlorellales_X | **ASV_150** | Cryomonadida | Cryothecomonas-lineage | **0.65** | 0 |
| **ASV_145** | Suessiales | Suessiaceae | **ASV_629** | Prymnesiales | Prymnesiophyceae_Clade_B5 | **0.65** | 0 |
| **ASV_842** | Dino-Group-I | Dino-Group-I-Clade-5 | **ASV_967** | Choreotrichida | Lynnellidae | **0.65** | 0 |
| **ASV_871** | Strombidiida | Strombidiidae_M | **ASV_900** | Pyramimonadales_X | Pyramimonadales_XX | **0.65** | 0 |
| **ASV_32** | Prorocentrales | Prorocentraceae | **ASV_195** | Dino-Group-I | Dino-Group-I-Clade-1 | **0.65** | 0 |
| **ASV_933** | Tintinnida | Eutintinnidae | **ASV_951** | Telonemia_XX | Telonemia-Group-2 | **0.65** | 0 |
| **ASV_607** | Dinophyceae_X | Dinophyceae_XX | **ASV_2086** | Strombidiida | Strombidiidae_Q | **0.65** | 0 |
| **ASV_842** | Dino-Group-I | Dino-Group-I-Clade-5 | **ASV_1054** | Telonemia_XX | Telonemia-Group-2 | **0.65** | 0 |
| **ASV_967** | Choreotrichida | Lynnellidae | **ASV_1062** | Bacillariophyta_X | Radial-centric-basal-Coscinodiscophyceae | **0.65** | 0 |
| **ASV_842** | Dino-Group-I | Dino-Group-I-Clade-5 | **ASV_898** | Dino-Group-II | Dino-Group-II-Clade-14 | **0.65** | 0 |
| **ASV_67** | Bacillariophyta_X | Polar-centric-Mediophyceae | **ASV_158** | Dinophyceae_X | Dinophyceae_XX | **0.65** | 0 |
| **ASV_951** | Telonemia_XX | Telonemia-Group-2 | **ASV_964** | Dino-Group-I | Dino-Group-I-Clade-1 | **0.65** | 0 |
| **ASV_1711** | Cryomonadida | Protaspa-lineage | **ASV_2160** | Dino-Group-I | Dino-Group-I-Clade-4 | **0.65** | 0 |
| **ASV_964** | Dino-Group-I | Dino-Group-I-Clade-1 | **ASV_1037** | Dinophyceae_X | Dinophyceae_XX | **0.65** | 0 |
| **ASV_35** | Chlorellales | Chlorellales_X | **ASV_78** | Gregarines | Cephaloidophoroidea | **0.65** | 0 |
| **ASV_26** | Bacillariophyta_X | Polar-centric-Mediophyceae | **ASV_281** | Bacillariophyta_X | Raphid-pennate | **0.65** | 0 |
| **ASV_937** | MAST-1 | MAST-1C | **ASV_961** | Dinophyceae_X | Dinophyceae_XX | **0.65** | 0 |
| **ASV_58** | Pelagomonadales | Pelagomonadaceae | **ASV_150** | Cryomonadida | Cryothecomonas-lineage | **0.65** | 0 |
| **ASV_962** | Bacillariophyta_X | Raphid-pennate | **ASV_1179** | Bacillariophyta_X | Raphid-pennate | **0.65** | 0 |
| **ASV_607** | Dinophyceae_X | Dinophyceae_XX | **ASV_845** | Crustacea | Maxillopoda | **0.65** | 0 |
| **ASV_2** | Bacillariophyta_X | Polar-centric-Mediophyceae | **ASV_22** | Pelagomonadales | Pelagomonadaceae | **0.65** | 0 |
| **ASV_866** | Chlamydomonadales | Chlamydomonadales_X | **ASV_959** | Chlorodendrales | Chlorodendraceae | **0.65** | 0 |
| **ASV_560** | Katablepharidales | Katablepharidales_X | **ASV_772** | Chlorarachnida | Chlorarachnida_X | **0.65** | 0 |
| **ASV_158** | Dinophyceae_X | Dinophyceae_XX | **ASV_669** | Dictyochophyceae_X | Dictyochales | **0.64** | 0 |
| **ASV_993** | MAST-1 | MAST-1C | **ASV_1038** | Mamiellales | Mamiellaceae | **0.64** | 0 |
| **ASV_15** | Mamiellales | Bathycoccaceae | **ASV_31** | Choreotrichida | Leegaardiellidae_B | **0.64** | 0 |
| **ASV_607** | Dinophyceae_X | Dinophyceae_XX | **ASV_900** | Pyramimonadales_X | Pyramimonadales_XX | **0.64** | 0 |
| **ASV_925** | Peridiniales | Protoperidiniaceae | **ASV_1255** | Peridiniales | Protoperidiniaceae | **0.64** | 0 |
| **ASV_15** | Mamiellales | Bathycoccaceae | **ASV_74** | Mamiellales | Mamiellaceae | **0.64** | 0 |
| **ASV_961** | Dinophyceae_X | Dinophyceae_XX | **ASV_1076** | Bacillariophyta_X | Radial-centric-basal-Coscinodiscophyceae | **0.64** | 0 |
| **ASV_967** | Choreotrichida | Lynnellidae | **ASV_1076** | Bacillariophyta_X | Radial-centric-basal-Coscinodiscophyceae | **0.64** | 0 |
| **ASV_230** | Telonemia_XX | Telonemia-Group-2 | **ASV_349** | Dinophyceae_X | Dinophyceae_XX | **0.64** | 0 |
| **ASV_900** | Pyramimonadales_X | Pyramimonadales_XX | **ASV_1062** | Bacillariophyta_X | Radial-centric-basal-Coscinodiscophyceae | **0.64** | 0 |
| **ASV_900** | Pyramimonadales_X | Pyramimonadales_XX | **ASV_1259** | Dinophyceae_X | Dinophyceae_XX | **0.64** | 0 |
| **ASV_26** | Bacillariophyta_X | Polar-centric-Mediophyceae | **ASV_237** | Dino-Group-II | Dino-Group-II-Clade-10-and-11 | **0.64** | 0 |
| **ASV_150** | Cryomonadida | Cryothecomonas-lineage | **ASV_559** | Cryptophyceae_X | Cryptomonadales | **0.64** | 0 |
| **ASV_772** | Chlorarachnida | Chlorarachnida_X | **ASV_929** | Dino-Group-II | Dino-Group-II-Clade-3 | **0.64** | 0 |
| **ASV_20** | Fungi_XX | Fungi_XXX | **ASV_34** | Pyramimonadales_X | Pyramimonadales_XX | **0.64** | 0 |
| **ASV_929** | Dino-Group-II | Dino-Group-II-Clade-3 | **ASV_945** | Chrysophyceae_X | Chrysophyceae_Clade-C | **0.64** | 0 |
| **ASV_1619** | Bacillariophyta_X | Raphid-pennate | **ASV_2044** | Collodaria | Collodaria_X | **0.64** | 0 |
| **ASV_2** | Bacillariophyta_X | Polar-centric-Mediophyceae | **ASV_237** | Dino-Group-II | Dino-Group-II-Clade-10-and-11 | **0.64** | 0 |
| **ASV_708** | Filosa-Thecofilosea_X | Mataza-lineage | **ASV_2204** | Dino-Group-I | Dino-Group-I-Clade-1 | **0.64** | 0 |
| **ASV_3** | Bacillariophyta_X | Polar-centric-Mediophyceae | **ASV_2204** | Dino-Group-I | Dino-Group-I-Clade-1 | **0.64** | 0 |
| **ASV_1035** | Dino-Group-I | Dino-Group-I-Clade-5 | **ASV_1093** | Dino-Group-II | Dino-Group-II-Clade-10-and-11 | **0.64** | 0 |
| **ASV_26** | Bacillariophyta_X | Polar-centric-Mediophyceae | **ASV_37** | Filosa-Thecofilosea_X | Mataza-lineage | **0.64** | 0 |
| **ASV_779** | Raphidophyceae_X | Raphidophyceae_XX | **ASV_880** | Dino-Group-I | Dino-Group-I-Clade-1 | **0.64** | 0 |
| **ASV_26** | Bacillariophyta_X | Polar-centric-Mediophyceae | **ASV_150** | Cryomonadida | Cryothecomonas-lineage | **0.64** | 0 |
| **ASV_779** | Raphidophyceae_X | Raphidophyceae_XX | **ASV_1013** | Dino-Group-II | Dino-Group-II-Clade-7 | **0.64** | 0 |
| **ASV_607** | Dinophyceae_X | Dinophyceae_XX | **ASV_1014** | Gymnodiniales | Gymnodiniaceae | **0.64** | 0 |
| **ASV_3** | Bacillariophyta_X | Polar-centric-Mediophyceae | **ASV_182** | Dino-Group-II | Dino-Group-II-Clade-44 | **0.64** | 0 |
| **ASV_36** | Bacillariophyta_X | Polar-centric-Mediophyceae | **ASV_163** | Dino-Group-I | Dino-Group-I-Clade-1 | **0.64** | 0 |
| **ASV_40** | Mamiellales | Mamiellaceae | **ASV_108** | Dino-Group-II | Dino-Group-II-Clade-6 | **0.64** | 0 |
| **ASV_1041** | MAST-3 | MAST-3E | **ASV_2086** | Strombidiida | Strombidiidae_Q | **0.64** | 0 |
| **ASV_13** | Strombidiida_B | Strombidiida_B_X | **ASV_47** | Strombidiida | Strombidiidae | **0.64** | 0 |
| **ASV_145** | Suessiales | Suessiaceae | **ASV_232** | Pelagophyceae_X | Pelagophyceae_XX | **0.64** | 0 |
| **ASV_482** | Chlorellales | Chlorellales_X | **ASV_1570** | Dinophyceae_X | Dinophyceae_XX | **0.64** | 0 |
| **ASV_83** | MAST-7 | MAST-7B | **ASV_91** | MAST-4 | MAST-4E | **0.64** | 0 |
| **ASV_900** | Pyramimonadales_X | Pyramimonadales_XX | **ASV_1054** | Telonemia_XX | Telonemia-Group-2 | **0.64** | 0 |
| **ASV_22** | Pelagomonadales | Pelagomonadaceae | **ASV_108** | Dino-Group-II | Dino-Group-II-Clade-6 | **0.64** | 0 |
| **ASV_900** | Pyramimonadales_X | Pyramimonadales_XX | **ASV_2086** | Strombidiida | Strombidiidae_Q | **0.64** | 0 |
| **ASV_3** | Bacillariophyta_X | Polar-centric-Mediophyceae | **ASV_78** | Gregarines | Cephaloidophoroidea | **0.64** | 0 |
| **ASV_908** | Gymnodiniales | Gymnodiniaceae | **ASV_933** | Tintinnida | Eutintinnidae | **0.64** | 0 |
| **ASV_937** | MAST-1 | MAST-1C | **ASV_1009** | Bacillariophyta_X | Raphid-pennate | **0.64** | 0 |
| **ASV_67** | Bacillariophyta_X | Polar-centric-Mediophyceae | **ASV_877** | Crustacea | Maxillopoda | **0.64** | 0 |
| **ASV_900** | Pyramimonadales_X | Pyramimonadales_XX | **ASV_1076** | Bacillariophyta_X | Radial-centric-basal-Coscinodiscophyceae | **0.64** | 0 |
| **ASV_28** | Bacillariophyta_X | Polar-centric-Mediophyceae | **ASV_329** | MAST-6 | MAST-6_X | **0.64** | 0 |
| **ASV_230** | Telonemia_XX | Telonemia-Group-2 | **ASV_563** | MAST-3 | MAST-3D | **0.64** | 0 |
| **ASV_58** | Pelagomonadales | Pelagomonadaceae | **ASV_294** | Peridiniales | Amphidiniopsidaceae | **0.63** | 0 |
| **ASV_3** | Bacillariophyta_X | Polar-centric-Mediophyceae | **ASV_708** | Filosa-Thecofilosea_X | Mataza-lineage | **0.63** | 0 |
| **ASV_211** | Telonemia_XX | Telonemia-Group-2 | **ASV_230** | Telonemia_XX | Telonemia-Group-2 | **0.63** | 0 |
| **ASV_47** | Strombidiida | Strombidiidae | **ASV_137** | Bacillariophyta_X | Raphid-pennate | **0.63** | 0 |
| **ASV_7** | Cryptophyceae_X | Cryptomonadales | **ASV_12** | Cryptophyceae_X | Cryptomonadales | **0.63** | 0 |
| **ASV_842** | Dino-Group-I | Dino-Group-I-Clade-5 | **ASV_848** | Strombidiida | Strombidiidae | **0.63** | 0 |
| **ASV_901** | Bacillariophyta_X | Polar-centric-Mediophyceae | **ASV_1014** | Gymnodiniales | Gymnodiniaceae | **0.63** | 0 |
| **ASV_924** | Pyramimonadales_X | Pyramimonadales_XX | **ASV_1104** | Haptophyta_Clade_HAP3_X | Haptophyta_Clade_HAP3_XX | **0.63** | 0 |
| **ASV_349** | Dinophyceae_X | Dinophyceae_XX | **ASV_563** | MAST-3 | MAST-3D | **0.63** | 0 |
| **ASV_951** | Telonemia_XX | Telonemia-Group-2 | **ASV_1041** | MAST-3 | MAST-3E | **0.63** | 0 |
| **ASV_629** | Prymnesiales | Prymnesiophyceae_Clade_B5 | **ASV_866** | Chlamydomonadales | Chlamydomonadales_X | **0.63** | 0 |
| **ASV_779** | Raphidophyceae_X | Raphidophyceae_XX | **ASV_1076** | Bacillariophyta_X | Radial-centric-basal-Coscinodiscophyceae | **0.63** | 0 |
| **ASV_3** | Bacillariophyta_X | Polar-centric-Mediophyceae | **ASV_32** | Prorocentrales | Prorocentraceae | **0.63** | 0 |
| **ASV_842** | Dino-Group-I | Dino-Group-I-Clade-5 | **ASV_1041** | MAST-3 | MAST-3E | **0.63** | 0 |
| **ASV_2** | Bacillariophyta_X | Polar-centric-Mediophyceae | **ASV_1711** | Cryomonadida | Protaspa-lineage | **0.63** | 0 |
| **ASV_1076** | Bacillariophyta_X | Radial-centric-basal-Coscinodiscophyceae | **ASV_1224** | Bacillariophyta_X | Araphid-pennate | **0.63** | 0 |
| **ASV_1037** | Dinophyceae_X | Dinophyceae_XX | **ASV_1041** | MAST-3 | MAST-3E | **0.63** | 0 |
| **ASV_182** | Dino-Group-II | Dino-Group-II-Clade-44 | **ASV_1711** | Cryomonadida | Protaspa-lineage | **0.63** | 0 |
| **ASV_35** | Chlorellales | Chlorellales_X | **ASV_36** | Bacillariophyta_X | Polar-centric-Mediophyceae | **0.63** | 0 |
| **ASV_983** | Chlorellales | Chlorellales_X | **ASV_1035** | Dino-Group-I | Dino-Group-I-Clade-5 | **0.63** | 0 |
| **ASV_961** | Dinophyceae_X | Dinophyceae_XX | **ASV_1054** | Telonemia_XX | Telonemia-Group-2 | **0.63** | 0 |
| **ASV_937** | MAST-1 | MAST-1C | **ASV_1014** | Gymnodiniales | Gymnodiniaceae | **0.63** | 0 |
| **ASV_40** | Mamiellales | Mamiellaceae | **ASV_202** | MAST-7 | MAST-7B | **0.63** | 0 |
| **ASV_11** | Dinophyceae_X | Dinophyceae_XX | **ASV_25** | Mamiellales | Bathycoccaceae | **0.63** | 0 |
| **ASV_924** | Pyramimonadales_X | Pyramimonadales_XX | **ASV_942** | Dino-Group-II | Dino-Group-II-Clade-10-and-11 | **0.63** | 0 |
| **ASV_28** | Bacillariophyta_X | Polar-centric-Mediophyceae | **ASV_32** | Prorocentrales | Prorocentraceae | **0.63** | 0 |
| **ASV_893** | Gonyaulacales | Goniodomataceae | **ASV_1054** | Telonemia_XX | Telonemia-Group-2 | **0.63** | 0 |
| **ASV_1014** | Gymnodiniales | Gymnodiniaceae | **ASV_1224** | Bacillariophyta_X | Araphid-pennate | **0.63** | 0 |
| **ASV_1146** | Crustacea | Maxillopoda | **ASV_1679** | Pelagomonadales | Pelagomonadaceae | **0.63** | 0 |
| **ASV_772** | Chlorarachnida | Chlorarachnida_X | **ASV_993** | MAST-1 | MAST-1C | **0.63** | 0 |
| **ASV_900** | Pyramimonadales_X | Pyramimonadales_XX | **ASV_901** | Bacillariophyta_X | Polar-centric-Mediophyceae | **0.63** | 0 |
| **ASV_560** | Katablepharidales | Katablepharidales_X | **ASV_629** | Prymnesiales | Prymnesiophyceae_Clade_B5 | **0.63** | 0 |
| **ASV_933** | Tintinnida | Eutintinnidae | **ASV_961** | Dinophyceae_X | Dinophyceae_XX | **0.63** | 0 |
| **ASV_36** | Bacillariophyta_X | Polar-centric-Mediophyceae | **ASV_149** | Pyramimonadales_X | Pyramimonadales_XX | **0.63** | 0 |
| **ASV_15** | Mamiellales | Bathycoccaceae | **ASV_137** | Bacillariophyta_X | Raphid-pennate | **0.63** | 0 |
| **ASV_28** | Bacillariophyta_X | Polar-centric-Mediophyceae | **ASV_592** | Dino-Group-II | Dino-Group-II-Clade-3 | **0.63** | 0 |
| **ASV_15** | Mamiellales | Bathycoccaceae | **ASV_482** | Chlorellales | Chlorellales_X | **0.63** | 0 |
| **ASV_35** | Chlorellales | Chlorellales_X | **ASV_708** | Filosa-Thecofilosea_X | Mataza-lineage | **0.63** | 0 |
| **ASV_1570** | Dinophyceae_X | Dinophyceae_XX | **ASV_1711** | Cryomonadida | Protaspa-lineage | **0.63** | 0 |
| **ASV_967** | Choreotrichida | Lynnellidae | **ASV_1037** | Dinophyceae_X | Dinophyceae_XX | **0.63** | 0 |
| **ASV_35** | Chlorellales | Chlorellales_X | **ASV_2204** | Dino-Group-I | Dino-Group-I-Clade-1 | **0.63** | 0 |
| **ASV_1146** | Crustacea | Maxillopoda | **ASV_1711** | Cryomonadida | Protaspa-lineage | **0.63** | 0 |
| **ASV_49** | Dino-Group-III | Dino-Group-III_X | **ASV_84** | Dinophyceae_X | Dinophyceae_XX | **0.63** | 0 |
| **ASV_46** | Strombidiida | Strombidiidae_M | **ASV_137** | Bacillariophyta_X | Raphid-pennate | **0.63** | 0 |
| **ASV_877** | Crustacea | Maxillopoda | **ASV_1013** | Dino-Group-II | Dino-Group-II-Clade-7 | **0.62** | 0 |
| **ASV_3** | Bacillariophyta_X | Polar-centric-Mediophyceae | **ASV_149** | Pyramimonadales_X | Pyramimonadales_XX | **0.62** | 0 |
| **ASV_28** | Bacillariophyta_X | Polar-centric-Mediophyceae | **ASV_31** | Choreotrichida | Leegaardiellidae_B | **0.62** | 0 |
| **ASV_232** | Pelagophyceae_X | Pelagophyceae_XX | **ASV_629** | Prymnesiales | Prymnesiophyceae_Clade_B5 | **0.62** | 0 |
| **ASV_866** | Chlamydomonadales | Chlamydomonadales_X | **ASV_944** | Dinophyceae_X | Dinophyceae_XX | **0.62** | 0 |
| **ASV_964** | Dino-Group-I | Dino-Group-I-Clade-1 | **ASV_1041** | MAST-3 | MAST-3E | **0.62** | 0 |
| **ASV_211** | Telonemia_XX | Telonemia-Group-2 | **ASV_563** | MAST-3 | MAST-3D | **0.62** | 0 |
| **ASV_14** | Dino-Group-III | Dino-Group-III_X | **ASV_133** | Dictyochophyceae_X | Dictyochales | **0.62** | 0 |
| **ASV_67** | Bacillariophyta_X | Polar-centric-Mediophyceae | **ASV_669** | Dictyochophyceae_X | Dictyochales | **0.62** | 0 |
| **ASV_950** | Syndiniales_X | Syndiniales_XX | **ASV_1041** | MAST-3 | MAST-3E | **0.62** | 0 |
| **ASV_951** | Telonemia_XX | Telonemia-Group-2 | **ASV_1076** | Bacillariophyta_X | Radial-centric-basal-Coscinodiscophyceae | **0.62** | 0 |
| **ASV_877** | Crustacea | Maxillopoda | **ASV_900** | Pyramimonadales_X | Pyramimonadales_XX | **0.62** | 0 |
| **ASV_1014** | Gymnodiniales | Gymnodiniaceae | **ASV_1077** | Oomycota_X | Oomycota_XX | **0.62** | 0 |
| **ASV_46** | Strombidiida | Strombidiidae_M | **ASV_482** | Chlorellales | Chlorellales_X | **0.62** | 0 |
| **ASV_7** | Cryptophyceae_X | Cryptomonadales | **ASV_149** | Pyramimonadales_X | Pyramimonadales_XX | **0.62** | 0 |
| **ASV_150** | Cryomonadida | Cryothecomonas-lineage | **ASV_195** | Dino-Group-I | Dino-Group-I-Clade-1 | **0.62** | 0 |
| **ASV_950** | Syndiniales_X | Syndiniales_XX | **ASV_1317** | Dino-Group-I | Dino-Group-I-Clade-1 | **0.62** | 0 |
| **ASV_2** | Bacillariophyta_X | Polar-centric-Mediophyceae | **ASV_78** | Gregarines | Cephaloidophoroidea | **0.62** | 0 |
| **ASV_884** | Prymnesiophyceae_X | Braarudosphaeraceae | **ASV_900** | Pyramimonadales_X | Pyramimonadales_XX | **0.62** | 0 |
| **ASV_11** | Dinophyceae_X | Dinophyceae_XX | **ASV_164** | Strombidiida | Strombidiidae_K | **0.62** | 0 |
| **ASV_202** | MAST-7 | MAST-7B | **ASV_218** | MAST-4 | MAST-4D | **0.62** | 0 |
| **ASV_1014** | Gymnodiniales | Gymnodiniaceae | **ASV_1028** | Choreotrichida | Strobilidiidae_I | **0.62** | 0 |
| **ASV_933** | Tintinnida | Eutintinnidae | **ASV_1037** | Dinophyceae_X | Dinophyceae_XX | **0.62** | 0 |
| **ASV_1037** | Dinophyceae_X | Dinophyceae_XX | **ASV_1062** | Bacillariophyta_X | Radial-centric-basal-Coscinodiscophyceae | **0.62** | 0 |
| **ASV_1619** | Bacillariophyta_X | Raphid-pennate | **ASV_1679** | Pelagomonadales | Pelagomonadaceae | **0.62** | 0 |
| **ASV_929** | Dino-Group-II | Dino-Group-II-Clade-3 | **ASV_1035** | Dino-Group-I | Dino-Group-I-Clade-5 | **0.62** | 0 |
| **ASV_22** | Pelagomonadales | Pelagomonadaceae | **ASV_25** | Mamiellales | Bathycoccaceae | **0.62** | 0 |
| **ASV_47** | Strombidiida | Strombidiidae | **ASV_150** | Cryomonadida | Cryothecomonas-lineage | **0.62** | 0 |
| **ASV_893** | Gonyaulacales | Goniodomataceae | **ASV_900** | Pyramimonadales_X | Pyramimonadales_XX | **0.62** | 0 |
| **ASV_772** | Chlorarachnida | Chlorarachnida_X | **ASV_931** | Filosa-Imbricatea_X | Novel-clade-2 | **0.62** | 0 |
| **ASV_933** | Tintinnida | Eutintinnidae | **ASV_1054** | Telonemia_XX | Telonemia-Group-2 | **0.62** | 0 |
| **ASV_137** | Bacillariophyta_X | Raphid-pennate | **ASV_559** | Cryptophyceae_X | Cryptomonadales | **0.62** | 0 |
| **ASV_15** | Mamiellales | Bathycoccaceae | **ASV_47** | Strombidiida | Strombidiidae | **0.62** | 0 |
| **ASV_2** | Bacillariophyta_X | Polar-centric-Mediophyceae | **ASV_93** | Katablepharidales | Katablepharidales_X | **0.62** | 0 |
| **ASV_57** | Dino-Group-II | Dino-Group-II-Clade-10-and-11 | **ASV_103** | Dino-Group-II | Dino-Group-II-Clade-26 | **0.62** | 0 |
| **ASV_1054** | Telonemia_XX | Telonemia-Group-2 | **ASV_1076** | Bacillariophyta_X | Radial-centric-basal-Coscinodiscophyceae | **0.62** | 0 |
| **ASV_2** | Bacillariophyta_X | Polar-centric-Mediophyceae | **ASV_61** | Mamiellales | Mamiellaceae | **0.62** | 0 |
| **ASV_866** | Chlamydomonadales | Chlamydomonadales_X | **ASV_912** | MOCH-2 | MOCH-2_X | **0.62** | 0 |
| **ASV_842** | Dino-Group-I | Dino-Group-I-Clade-5 | **ASV_996** | Dino-Group-II | Dino-Group-II-Clade-5 | **0.62** | 0 |
| **ASV_482** | Chlorellales | Chlorellales_X | **ASV_530** | MAST-12 | MAST-12A | **0.62** | 0 |
| **ASV_1539** | Cyclotrichia | Mesodiniidae | **ASV_2086** | Strombidiida | Strombidiidae_Q | **0.62** | 0 |
| **ASV_2** | Bacillariophyta_X | Polar-centric-Mediophyceae | **ASV_163** | Dino-Group-I | Dino-Group-I-Clade-1 | **0.62** | 0 |
| **ASV_47** | Strombidiida | Strombidiidae | **ASV_386** | Cryomonadida | Protaspa-lineage | **0.62** | 0 |
| **ASV_772** | Chlorarachnida | Chlorarachnida_X | **ASV_1038** | Mamiellales | Mamiellaceae | **0.62** | 0 |
| **ASV_15** | Mamiellales | Bathycoccaceae | **ASV_91** | MAST-4 | MAST-4E | **0.62** | 0 |
| **ASV_93** | Katablepharidales | Katablepharidales_X | **ASV_137** | Bacillariophyta_X | Raphid-pennate | **0.62** | 0 |
| **ASV_779** | Raphidophyceae_X | Raphidophyceae_XX | **ASV_1271** | Dino-Group-II | Dino-Group-II-Clade-7 | **0.62** | 0 |
| **ASV_15** | Mamiellales | Bathycoccaceae | **ASV_202** | MAST-7 | MAST-7B | **0.62** | 0 |
| **ASV_1014** | Gymnodiniales | Gymnodiniaceae | **ASV_1259** | Dinophyceae_X | Dinophyceae_XX | **0.62** | 0 |
| **ASV_47** | Strombidiida | Strombidiidae | **ASV_93** | Katablepharidales | Katablepharidales_X | **0.62** | 0 |
| **ASV_40** | Mamiellales | Mamiellaceae | **ASV_218** | MAST-4 | MAST-4D | **0.62** | 0 |
| **ASV_2** | Bacillariophyta_X | Polar-centric-Mediophyceae | **ASV_31** | Choreotrichida | Leegaardiellidae_B | **0.62** | 0 |
| **ASV_560** | Katablepharidales | Katablepharidales_X | **ASV_586** | Dino-Group-II | Dino-Group-II_X | **0.62** | 0 |
| **ASV_772** | Chlorarachnida | Chlorarachnida_X | **ASV_866** | Chlamydomonadales | Chlamydomonadales_X | **0.62** | 0 |
| **ASV_607** | Dinophyceae_X | Dinophyceae_XX | **ASV_1317** | Dino-Group-I | Dino-Group-I-Clade-1 | **0.62** | 0 |
| **ASV_4** | Cryptophyceae_X | Cryptomonadales | **ASV_12** | Cryptophyceae_X | Cryptomonadales | **0.62** | 0 |
| **ASV_4** | Cryptophyceae_X | Cryptomonadales | **ASV_149** | Pyramimonadales_X | Pyramimonadales_XX | **0.62** | 0 |
| **ASV_933** | Tintinnida | Eutintinnidae | **ASV_1271** | Dino-Group-II | Dino-Group-II-Clade-7 | **0.61** | 0 |
| **ASV_137** | Bacillariophyta_X | Raphid-pennate | **ASV_309** | Bacillariophyta_X | Raphid-pennate | **0.61** | 0 |
| **ASV_1020** | Phaeocystales | Phaeocystaceae | **ASV_1057** | Dino-Group-II | Dino-Group-II-Clade-32 | **0.61** | 0 |
| **ASV_67** | Bacillariophyta_X | Polar-centric-Mediophyceae | **ASV_1037** | Dinophyceae_X | Dinophyceae_XX | **0.61** | 0 |
| **ASV_31** | Choreotrichida | Leegaardiellidae_B | **ASV_57** | Dino-Group-II | Dino-Group-II-Clade-10-and-11 | **0.61** | 0 |
| **ASV_607** | Dinophyceae_X | Dinophyceae_XX | **ASV_1057** | Dino-Group-II | Dino-Group-II-Clade-32 | **0.61** | 0 |
| **ASV_57** | Dino-Group-II | Dino-Group-II-Clade-10-and-11 | **ASV_195** | Dino-Group-I | Dino-Group-I-Clade-1 | **0.61** | 0 |
| **ASV_1054** | Telonemia_XX | Telonemia-Group-2 | **ASV_1812** | Noctilucales | Noctilucaceae | **0.61** | 0 |
| **ASV_893** | Gonyaulacales | Goniodomataceae | **ASV_951** | Telonemia_XX | Telonemia-Group-2 | **0.61** | 0 |
| **ASV_93** | Katablepharidales | Katablepharidales_X | **ASV_559** | Cryptophyceae_X | Cryptomonadales | **0.61** | 0 |
| **ASV_607** | Dinophyceae_X | Dinophyceae_XX | **ASV_1077** | Oomycota_X | Oomycota_XX | **0.61** | 0 |
| **ASV_26** | Bacillariophyta_X | Polar-centric-Mediophyceae | **ASV_708** | Filosa-Thecofilosea_X | Mataza-lineage | **0.61** | 0 |
| **ASV_108** | Dino-Group-II | Dino-Group-II-Clade-6 | **ASV_218** | MAST-4 | MAST-4D | **0.61** | 0 |
| **ASV_1014** | Gymnodiniales | Gymnodiniaceae | **ASV_1054** | Telonemia_XX | Telonemia-Group-2 | **0.61** | 0 |
| **ASV_951** | Telonemia_XX | Telonemia-Group-2 | **ASV_1317** | Dino-Group-I | Dino-Group-I-Clade-1 | **0.61** | 0 |
| **ASV_772** | Chlorarachnida | Chlorarachnida_X | **ASV_940** | Telonemia_XX | Telonemia-Group-1 | **0.61** | 0 |
| **ASV_137** | Bacillariophyta_X | Raphid-pennate | **ASV_150** | Cryomonadida | Cryothecomonas-lineage | **0.61** | 0 |
| **ASV_67** | Bacillariophyta_X | Polar-centric-Mediophyceae | **ASV_1062** | Bacillariophyta_X | Radial-centric-basal-Coscinodiscophyceae | **0.61** | 0 |
| **ASV_373** | Dinophyceae_X | Dinophyceae_XX | **ASV_586** | Dino-Group-II | Dino-Group-II_X | **0.61** | 0 |
| **ASV_15** | Mamiellales | Bathycoccaceae | **ASV_68** | Mamiellales | Mamiellaceae | **0.61** | 0 |
| **ASV_942** | Dino-Group-II | Dino-Group-II-Clade-10-and-11 | **ASV_993** | MAST-1 | MAST-1C | **0.61** | 0 |
| **ASV_779** | Raphidophyceae_X | Raphidophyceae_XX | **ASV_1062** | Bacillariophyta_X | Radial-centric-basal-Coscinodiscophyceae | **0.61** | 0 |
| **ASV_933** | Tintinnida | Eutintinnidae | **ASV_1014** | Gymnodiniales | Gymnodiniaceae | **0.61** | 0 |
| **ASV_31** | Choreotrichida | Leegaardiellidae_B | **ASV_150** | Cryomonadida | Cryothecomonas-lineage | **0.61** | 0 |
| **ASV_34** | Pyramimonadales_X | Pyramimonadales_XX | **ASV_118** | Phaeocystales | Phaeocystaceae | **0.61** | 0 |
| **ASV_329** | MAST-6 | MAST-6_X | **ASV_482** | Chlorellales | Chlorellales_X | **0.61** | 0 |
| **ASV_900** | Pyramimonadales_X | Pyramimonadales_XX | **ASV_933** | Tintinnida | Eutintinnidae | **0.61** | 0 |
| **ASV_858** | Bacillariophyta_X | Polar-centric-Mediophyceae | **ASV_859** | Bacillariophyta_X | Polar-centric-Mediophyceae | **0.61** | 0 |
| **ASV_2** | Bacillariophyta_X | Polar-centric-Mediophyceae | **ASV_59** | MAST-3 | MAST-3E | **0.61** | 0 |
| **ASV_281** | Bacillariophyta_X | Raphid-pennate | **ASV_328** | Dino-Group-II | Dino-Group-II-Clade-21 | **0.61** | 0 |
| **ASV_294** | Peridiniales | Amphidiniopsidaceae | **ASV_1711** | Cryomonadida | Protaspa-lineage | **0.61** | 0 |
| **ASV_2** | Bacillariophyta_X | Polar-centric-Mediophyceae | **ASV_57** | Dino-Group-II | Dino-Group-II-Clade-10-and-11 | **0.61** | 0 |
| **ASV_961** | Dinophyceae_X | Dinophyceae_XX | **ASV_1077** | Oomycota_X | Oomycota_XX | **0.61** | 0 |
| **ASV_929** | Dino-Group-II | Dino-Group-II-Clade-3 | **ASV_983** | Chlorellales | Chlorellales_X | **0.61** | 0 |
| **ASV_1009** | Bacillariophyta_X | Raphid-pennate | **ASV_1062** | Bacillariophyta_X | Radial-centric-basal-Coscinodiscophyceae | **0.61** | 0 |
| **ASV_3** | Bacillariophyta_X | Polar-centric-Mediophyceae | **ASV_2160** | Dino-Group-I | Dino-Group-I-Clade-4 | **0.61** | 0 |
| **ASV_937** | MAST-1 | MAST-1C | **ASV_967** | Choreotrichida | Lynnellidae | **0.61** | 0 |
| **ASV_951** | Telonemia_XX | Telonemia-Group-2 | **ASV_1062** | Bacillariophyta_X | Radial-centric-basal-Coscinodiscophyceae | **0.61** | 0 |
| **ASV_2** | Bacillariophyta_X | Polar-centric-Mediophyceae | **ASV_32** | Prorocentrales | Prorocentraceae | **0.61** | 0 |
| **ASV_951** | Telonemia_XX | Telonemia-Group-2 | **ASV_2086** | Strombidiida | Strombidiidae_Q | **0.61** | 0 |
| **ASV_983** | Chlorellales | Chlorellales_X | **ASV_993** | MAST-1 | MAST-1C | **0.61** | 0 |
| **ASV_57** | Dino-Group-II | Dino-Group-II-Clade-10-and-11 | **ASV_91** | MAST-4 | MAST-4E | **0.61** | 0 |
| **ASV_11** | Dinophyceae_X | Dinophyceae_XX | **ASV_109** | Dino-Group-II | Dino-Group-II-Clade-6 | **0.61** | 0 |
| **ASV_58** | Pelagomonadales | Pelagomonadaceae | **ASV_237** | Dino-Group-II | Dino-Group-II-Clade-10-and-11 | **0.61** | 0 |
| **ASV_837** | Chlorarachnida | Chlorarachnida_X | **ASV_880** | Dino-Group-I | Dino-Group-I-Clade-1 | **0.61** | 0 |
| **ASV_67** | Bacillariophyta_X | Polar-centric-Mediophyceae | **ASV_842** | Dino-Group-I | Dino-Group-I-Clade-5 | **0.61** | 0 |
| **ASV_150** | Cryomonadida | Cryothecomonas-lineage | **ASV_237** | Dino-Group-II | Dino-Group-II-Clade-10-and-11 | **0.61** | 0 |
| **ASV_204** | Pezizomycotina | New-Pezizomycotina-family | **ASV_249** | Dino-Group-II | Dino-Group-II-Clade-10-and-11 | **0.61** | 0 |
| **ASV_3** | Bacillariophyta_X | Polar-centric-Mediophyceae | **ASV_84** | Dinophyceae_X | Dinophyceae_XX | **0.61** | 0 |
| **ASV_31** | Choreotrichida | Leegaardiellidae_B | **ASV_124** | Cyclotrichia | Cyclotrichia_X | **0.61** | 0 |
| **ASV_900** | Pyramimonadales_X | Pyramimonadales_XX | **ASV_1539** | Cyclotrichia | Mesodiniidae | **0.61** | 0 |
| **ASV_924** | Pyramimonadales_X | Pyramimonadales_XX | **ASV_993** | MAST-1 | MAST-1C | **0.61** | 0 |
| **ASV_1037** | Dinophyceae_X | Dinophyceae_XX | **ASV_1077** | Oomycota_X | Oomycota_XX | **0.61** | 0 |
| **ASV_158** | Dinophyceae_X | Dinophyceae_XX | **ASV_779** | Raphidophyceae_X | Raphidophyceae_XX | **0.61** | 0 |
| **ASV_25** | Mamiellales | Bathycoccaceae | **ASV_109** | Dino-Group-II | Dino-Group-II-Clade-6 | **0.61** | 0 |
| **ASV_137** | Bacillariophyta_X | Raphid-pennate | **ASV_249** | Dino-Group-II | Dino-Group-II-Clade-10-and-11 | **0.61** | 0 |
| **ASV_28** | Bacillariophyta_X | Polar-centric-Mediophyceae | **ASV_309** | Bacillariophyta_X | Raphid-pennate | **0.61** | 0 |
| **ASV_832** | Dinophyceae_X | Dinophyceae_XX | **ASV_1014** | Gymnodiniales | Gymnodiniaceae | **0.61** | 0 |
| **ASV_877** | Crustacea | Maxillopoda | **ASV_967** | Choreotrichida | Lynnellidae | **0.61** | 0 |
| **ASV_328** | Dino-Group-II | Dino-Group-II-Clade-21 | **ASV_482** | Chlorellales | Chlorellales_X | **0.61** | 0 |
| **ASV_867** | Dinophyceae_X | Dinophyceae_XX | **ASV_944** | Dinophyceae_X | Dinophyceae_XX | **0.61** | 0 |
| **ASV_103** | Dino-Group-II | Dino-Group-II-Clade-26 | **ASV_291** | Dino-Group-II | Dino-Group-II-Clade-10-and-11 | **0.61** | 0 |
| **ASV_95** | Prymnesiales | Prymnesiaceae | **ASV_707** | Dino-Group-I | Dino-Group-I-Clade-1 | **0.61** | 0 |
| **ASV_607** | Dinophyceae_X | Dinophyceae_XX | **ASV_1054** | Telonemia_XX | Telonemia-Group-2 | **0.61** | 0 |
| **ASV_35** | Chlorellales | Chlorellales_X | **ASV_2160** | Dino-Group-I | Dino-Group-I-Clade-4 | **0.61** | 0 |
| **ASV_137** | Bacillariophyta_X | Raphid-pennate | **ASV_329** | MAST-6 | MAST-6_X | **0.61** | 0 |
| **ASV_25** | Mamiellales | Bathycoccaceae | **ASV_171** | Katablepharidales | Katablepharidales_X | **0.61** | 0 |
| **ASV_15** | Mamiellales | Bathycoccaceae | **ASV_108** | Dino-Group-II | Dino-Group-II-Clade-6 | **0.61** | 0 |
| **ASV_779** | Raphidophyceae_X | Raphidophyceae_XX | **ASV_924** | Pyramimonadales_X | Pyramimonadales_XX | **0.61** | 0 |
| **ASV_937** | MAST-1 | MAST-1C | **ASV_1062** | Bacillariophyta_X | Radial-centric-basal-Coscinodiscophyceae | **0.60** | 0 |
| **ASV_36** | Bacillariophyta_X | Polar-centric-Mediophyceae | **ASV_1711** | Cryomonadida | Protaspa-lineage | **0.60** | 0 |
| **ASV_779** | Raphidophyceae_X | Raphidophyceae_XX | **ASV_908** | Gymnodiniales | Gymnodiniaceae | **0.60** | 0 |
| **ASV_961** | Dinophyceae_X | Dinophyceae_XX | **ASV_1224** | Bacillariophyta_X | Araphid-pennate | **0.60** | 0 |
| **ASV_1224** | Bacillariophyta_X | Araphid-pennate | **ASV_1259** | Dinophyceae_X | Dinophyceae_XX | **0.60** | 0 |
| **ASV_1679** | Pelagomonadales | Pelagomonadaceae | **ASV_2133** | Dinophyceae_X | Dinophyceae_XX | **0.60** | 0 |
| **ASV_779** | Raphidophyceae_X | Raphidophyceae_XX | **ASV_1812** | Noctilucales | Noctilucaceae | **0.60** | 0 |
| **ASV_67** | Bacillariophyta_X | Polar-centric-Mediophyceae | **ASV_961** | Dinophyceae_X | Dinophyceae_XX | **0.60** | 0 |
| **ASV_2** | Bacillariophyta_X | Polar-centric-Mediophyceae | **ASV_84** | Dinophyceae_X | Dinophyceae_XX | **0.60** | 0 |
| **ASV_933** | Tintinnida | Eutintinnidae | **ASV_1041** | MAST-3 | MAST-3E | **0.60** | 0 |
| **ASV_40** | Mamiellales | Mamiellaceae | **ASV_58** | Pelagomonadales | Pelagomonadaceae | **0.60** | 0 |
| **ASV_893** | Gonyaulacales | Goniodomataceae | **ASV_1062** | Bacillariophyta_X | Radial-centric-basal-Coscinodiscophyceae | **0.60** | 0 |
| **ASV_3** | Bacillariophyta_X | Polar-centric-Mediophyceae | **ASV_37** | Filosa-Thecofilosea_X | Mataza-lineage | **0.60** | 0 |
| **ASV_950** | Syndiniales_X | Syndiniales_XX | **ASV_1077** | Oomycota_X | Oomycota_XX | **0.60** | 0 |
| **ASV_203** | Filosa-Imbricatea_X | Novel-clade-2 | **ASV_779** | Raphidophyceae_X | Raphidophyceae_XX | **0.60** | 0 |
| **ASV_877** | Crustacea | Maxillopoda | **ASV_901** | Bacillariophyta_X | Polar-centric-Mediophyceae | **0.60** | 0 |
| **ASV_39** | Tintinnida | Stenosemellidae | **ASV_386** | Cryomonadida | Protaspa-lineage | **0.60** | 0 |
| **ASV_31** | Choreotrichida | Leegaardiellidae_B | **ASV_47** | Strombidiida | Strombidiidae | **0.60** | 0 |
| **ASV_145** | Suessiales | Suessiaceae | **ASV_373** | Dinophyceae_X | Dinophyceae_XX | **0.60** | 0 |
| **ASV_12** | Cryptophyceae_X | Cryptomonadales | **ASV_15** | Mamiellales | Bathycoccaceae | **0.60** | 0 |
| **ASV_227** | Chlorodendrales | Chlorodendraceae | **ASV_232** | Pelagophyceae_X | Pelagophyceae_XX | **0.60** | 0 |
| **ASV_779** | Raphidophyceae_X | Raphidophyceae_XX | **ASV_1317** | Dino-Group-I | Dino-Group-I-Clade-1 | **0.60** | 0 |
| **ASV_26** | Bacillariophyta_X | Polar-centric-Mediophyceae | **ASV_573** | Bacillariophyta_X | Araphid-pennate | **0.60** | 0 |
| **ASV_39** | Tintinnida | Stenosemellidae | **ASV_137** | Bacillariophyta_X | Raphid-pennate | **0.60** | 0 |
| **ASV_845** | Crustacea | Maxillopoda | **ASV_933** | Tintinnida | Eutintinnidae | **0.60** | 0 |
| **ASV_26** | Bacillariophyta_X | Polar-centric-Mediophyceae | **ASV_149** | Pyramimonadales_X | Pyramimonadales_XX | **0.60** | 0 |
| **ASV_1009** | Bacillariophyta_X | Raphid-pennate | **ASV_1014** | Gymnodiniales | Gymnodiniaceae | **0.60** | 0 |
| **ASV_28** | Bacillariophyta_X | Polar-centric-Mediophyceae | **ASV_84** | Dinophyceae_X | Dinophyceae_XX | **0.60** | 0 |
| **ASV_1045** | Bacillariophyta_X | Radial-centric-basal-Coscinodiscophyceae | **ASV_1062** | Bacillariophyta_X | Radial-centric-basal-Coscinodiscophyceae | **0.60** | 0 |
| **ASV_973** | Dino-Group-II | Dino-Group-II-Clade-4 | **ASV_1443** | Chrysophyceae_X | Chrysophyceae_Clade-E | **0.60** | 0 |
| **ASV_951** | Telonemia_XX | Telonemia-Group-2 | **ASV_986** | Prorocentrales | Prorocentraceae | **0.60** | 0 |
| **ASV_281** | Bacillariophyta_X | Raphid-pennate | **ASV_294** | Peridiniales | Amphidiniopsidaceae | **0.60** | 0 |
| **ASV_563** | MAST-3 | MAST-3D | **ASV_1146** | Crustacea | Maxillopoda | **0.60** | 0 |
| **ASV_276** | Prymnesiales | Prymnesiophyceae_Clade_B4 | **ASV_560** | Katablepharidales | Katablepharidales_X | **0.60** | 0 |
| **ASV_961** | Dinophyceae_X | Dinophyceae_XX | **ASV_1013** | Dino-Group-II | Dino-Group-II-Clade-7 | **0.60** | 0 |
| **ASV_1009** | Bacillariophyta_X | Raphid-pennate | **ASV_1224** | Bacillariophyta_X | Araphid-pennate | **0.60** | 0 |
